# Test of CP invariance in vector-boson fusion production of the Higgs boson using the *Optimal Observable* method in the ditau decay channel with the ATLAS detector

**DOI:** 10.1140/epjc/s10052-016-4499-5

**Published:** 2016-11-29

**Authors:** G. Aad, B. Abbott, O. Abdinov, J. Abdallah, B. Abeloos, R. Aben, M. Abolins, R. Aben, M. Abolins, O. S. AbouZeid, N. L. Abraham, H. Abramowicz, H. Abreu, R. Abreu, Y. Abulaiti, B. S. Acharya, L. Adamczyk, D. L. Adams, J. Adelman, S. Adomeit, T. Adye, A. A. Affolder, T. Agatonovic-Jovin, J. Agricola, J. A. Aguilar-Saavedra, S. P. Ahlen, F. Ahmadov, G. Aielli, H. Akerstedt, T. P. A. Åkesson, A. V. Akimov, G. L. Alberghi, J. Albert, S. Albrand, M. J. Alconada Verzini, M. Aleksa, I. N. Aleksandrov, C. Alexa, G. Alexander, T. Alexopoulos, M. Alhroob, G. Alimonti, J. Alison, S. P. Alkire, B. M. M. Allbrooke, B. W. Allen, P. P. Allport, A. Aloisio, A. Alonso, F. Alonso, C. Alpigiani, B. Alvarez Gonzalez, D. Álvarez Piqueras, M. G. Alviggi, B. T. Amadio, K. Amako, Y. Amaral Coutinho, C. Amelung, D. Amidei, S. P. Amor Dos Santos, A. Amorim, S. Amoroso, N. Amram, G. Amundsen, C. Anastopoulos, L. S. Ancu, N. Andari, T. Andeen, C. F. Anders, G. Anders, J. K. Anders, K. J. Anderson, A. Andreazza, V. Andrei, S. Angelidakis, I. Angelozzi, P. Anger, A. Angerami, F. Anghinolfi, A. V. Anisenkov, N. Anjos, A. Annovi, M. Antonelli, A. Antonov, J. Antos, F. Anulli, M. Aoki, L. Aperio Bella, G. Arabidze, Y. Arai, J. P. Araque, A. T. H. Arce, F. A. Arduh, J.-F. Arguin, S. Argyropoulos, M. Arik, A. J. Armbruster, L. J. Armitage, O. Arnaez, H. Arnold, M. Arratia, O. Arslan, A. Artamonov, G. Artoni, S. Artz, S. Asai, N. Asbah, A. Ashkenazi, B. Åsman, L. Asquith, K. Assamagan, R. Astalos, M. Atkinson, N. B. Atlay, K. Augsten, G. Avolio, B. Axen, M. K. Ayoub, G. Azuelos, M. A. Baak, A. E. Baas, M. J. Baca, H. Bachacou, K. Bachas, M. Backes, M. Backhaus, P. Bagiacchi, P. Bagnaia, Y. Bai, J. T. Baines, O. K. Baker, E. M. Baldin, P. Balek, T. Balestri, F. Balli, W. K. Balunas, E. Banas, Sw. Banerjee, A. A. E. Bannoura, L. Barak, E. L. Barberio, D. Barberis, M. Barbero, T. Barillari, M. Barisonzi, T. Barklow, N. Barlow, S. L. Barnes, B. M. Barnett, R. M. Barnett, Z. Barnovska, A. Baroncelli, G. Barone, A. J. Barr, L. Barranco Navarro, F. Barreiro, J. Barreiro Guimarães da Costa, R. Bartoldus, A. E. Barton, P. Bartos, A. Basalaev, A. Bassalat, A. Basye, R. L. Bates, S. J. Batista, J. R. Batley, M. Battaglia, M. Bauce, F. Bauer, H. S. Bawa, J. B. Beacham, M. D. Beattie, T. Beau, P. H. Beauchemin, P. Bechtle, H. P. Beck, K. Becker, M. Becker, M. Beckingham, C. Becot, A. J. Beddall, A. Beddall, V. A. Bednyakov, M. Bedognetti, C. P. Bee, L. J. Beemster, T. A. Beermann, M. Begel, J. K. Behr, C. Belanger-Champagne, A. S. Bell, W. H. Bell, G. Bella, L. Bellagamba, A. Bellerive, M. Bellomo, K. Belotskiy, O. Beltramello, N. L. Belyaev, O. Benary, D. Benchekroun, M. Bender, K. Bendtz, N. Benekos, Y. Benhammou, E. Benhar Noccioli, J. Benitez, J. A. Benitez Garcia, D. P. Benjamin, J. R. Bensinger, S. Bentvelsen, L. Beresford, M. Beretta, D. Berge, E. Bergeaas Kuutmann, N. Berger, F. Berghaus, J. Beringer, S. Berlendis, N. R. Bernard, C. Bernius, F. U. Bernlochner, T. Berry, P. Berta, C. Bertella, G. Bertoli, F. Bertolucci, I. A. Bertram, C. Bertsche, D. Bertsche, G. J. Besjes, O. Bessidskaia Bylund, M. Bessner, N. Besson, C. Betancourt, S. Bethke, A. J. Bevan, W. Bhimji, R. M. Bianchi, L. Bianchini, M. Bianco, O. Biebel, D. Biedermann, R. Bielski, N. V. Biesuz, M. Biglietti, J. Bilbao De Mendizabal, H. Bilokon, M. Bindi, S. Binet, A. Bingul, C. Bini, S. Biondi, D. M. Bjergaard, C. W. Black, J. E. Black, K. M. Black, D. Blackburn, R. E. Blair, J.-B. Blanchard, J. E. Blanco, T. Blazek, I. Bloch, C. Blocker, W. Blum, U. Blumenschein, S. Blunier, G. J. Bobbink, V. S. Bobrovnikov, S. S. Bocchetta, A. Bocci, C. Bock, M. Boehler, D. Boerner, J. A. Bogaerts, D. Bogavac, A. G. Bogdanchikov, C. Bohm, V. Boisvert, T. Bold, V. Boldea, A. S. Boldyrev, M. Bomben, M. Bona, M. Boonekamp, A. Borisov, G. Borissov, J. Bortfeldt, D. Bortoletto, V. Bortolotto, K. Bos, D. Boscherini, M. Bosman, J. D. Bossio Sola, J. Boudreau, J. Bouffard, E. V. Bouhova-Thacker, D. Boumediene, C. Bourdarios, S. K. Boutle, A. Boveia, J. Boyd, I. R. Boyko, J. Bracinik, A. Brandt, G. Brandt, O. Brandt, U. Bratzler, B. Brau, J. E. Brau, H. M. Braun, W. D. Breaden Madden, K. Brendlinger, A. J. Brennan, L. Brenner, R. Brenner, S. Bressler, T. M. Bristow, D. Britton, D. Britzger, F. M. Brochu, I. Brock, R. Brock, G. Brooijmans, T. Brooks, W. K. Brooks, J. Brosamer, E. Brost, J. H Broughton, P. A. Bruckman de Renstrom, D. Bruncko, R. Bruneliere, A. Bruni, G. Bruni, B. H. Brunt, M. Bruschi, N. Bruscino, P. Bryant, L. Bryngemark, T. Buanes, Q. Buat, P. Buchholz, A. G. Buckley, I. A. Budagov, F. Buehrer, M. K. Bugge, O. Bulekov, D. Bullock, H. Burckhart, S. Burdin, C. D. Burgard, B. Burghgrave, K. Burka, S. Burke, I. Burmeister, E. Busato, D. Büscher, V. Büscher, P. Bussey, J. M. Butler, A. I. Butt, C. M. Buttar, J. M. Butterworth, P. Butti, W. Buttinger, A. Buzatu, A. R. Buzykaev, S. Cabrera Urbán, D. Caforio, V. M. Cairo, O. Cakir, N. Calace, P. Calafiura, A. Calandri, G. Calderini, P. Calfayan, L. P. Caloba, D. Calvet, S. Calvet, T. P. Calvet, R. Camacho Toro, S. Camarda, P. Camarri, D. Cameron, R. Caminal Armadans, C. Camincher, S. Campana, M. Campanelli, A. Campoverde, V. Canale, A. Canepa, M. Cano Bret, J. Cantero, R. Cantrill, T. Cao, M. D. M. Capeans Garrido, I. Caprini, M. Caprini, M. Capua, R. Caputo, R. M. Carbone, R. Cardarelli, F. Cardillo, T. Carli, G. Carlino, L. Carminati, S. Caron, E. Carquin, G. D. Carrillo-Montoya, J. R. Carter, J. Carvalho, D. Casadei, M. P. Casado, M. Casolino, D. W. Casper, E. Castaneda-Miranda, A. Castelli, V. Castillo Gimenez, N. F. Castro, A. Catinaccio, J. R. Catmore, A. Cattai, J. Caudron, V. Cavaliere, E. Cavallaro, D. Cavalli, M. Cavalli-Sforza, V. Cavasinni, F. Ceradini, L. Cerda Alberich, B. C. Cerio, A. S. Cerqueira, A. Cerri, L. Cerrito, F. Cerutti, M. Cerv, A. Cervelli, S. A. Cetin, A. Chafaq, D. Chakraborty, I. Chalupkova, S. K. Chan, Y. L. Chan, P. Chang, J. D. Chapman, D. G. Charlton, A. Chatterjee, C. C. Chau, C. A. Chavez Barajas, S. Che, S. Cheatham, A. Chegwidden, S. Chekanov, S. V. Chekulaev, G. A. Chelkov, M. A. Chelstowska, C. Chen, H. Chen, K. Chen, S. Chen, S. Chen, X. Chen, Y. Chen, H. C. Cheng, H. J Cheng, Y. Cheng, A. Cheplakov, E. Cheremushkina, R. Cherkaoui El Moursli, V. Chernyatin, E. Cheu, L. Chevalier, V. Chiarella, G. Chiarelli, G. Chiodini, A. S. Chisholm, A. Chitan, M. V. Chizhov, K. Choi, A. R. Chomont, S. Chouridou, B. K. B. Chow, V. Christodoulou, D. Chromek-Burckhart, J. Chudoba, A. J. Chuinard, J. J. Chwastowski, L. Chytka, G. Ciapetti, A. K. Ciftci, D. Cinca, V. Cindro, I. A. Cioara, A. Ciocio, F. Cirotto, Z. H. Citron, M. Ciubancan, A. Clark, B. L. Clark, P. J. Clark, R. N. Clarke, C. Clement, Y. Coadou, M. Cobal, A. Coccaro, J. Cochran, L. Coffey, L. Colasurdo, B. Cole, S. Cole, A. P. Colijn, J. Collot, T. Colombo, G. Compostella, P. Conde Muiño, E. Coniavitis, S. H. Connell, I. A. Connelly, V. Consorti, S. Constantinescu, C. Conta, G. Conti, F. Conventi, M. Cooke, B. D. Cooper, A. M. Cooper-Sarkar, T. Cornelissen, M. Corradi, F. Corriveau, A. Corso-Radu, A. Cortes-Gonzalez, G. Cortiana, G. Costa, M. J. Costa, D. Costanzo, G. Cottin, G. Cowan, B. E. Cox, K. Cranmer, S. J. Crawley, G. Cree, S. Crépé-Renaudin, F. Crescioli, W. A. Cribbs, M. Crispin Ortuzar, M. Cristinziani, V. Croft, G. Crosetti, T. Cuhadar Donszelmann, J. Cummings, M. Curatolo, J. Cúth, C. Cuthbert, H. Czirr, P. Czodrowski, S. D’Auria, M. D’Onofrio, M. J. Da Cunha Sargedas De Sousa, C. Da Via, W. Dabrowski, T. Dai, O. Dale, F. Dallaire, C. Dallapiccola, M. Dam, J. R. Dandoy, N. P. Dang, A. C. Daniells, N. S. Dann, M. Danninger, M. Dano Hoffmann, V. Dao, G. Darbo, S. Darmora, J. Dassoulas, A. Dattagupta, W. Davey, C. David, T. Davidek, M. Davies, P. Davison, Y. Davygora, E. Dawe, I. Dawson, R. K. Daya-Ishmukhametova, K. De, R. de Asmundis, A. De Benedetti, S. De Castro, S. De Cecco, N. De Groot, P. de Jong, H. De la Torre, F. De Lorenzi, D. De Pedis, A. De Salvo, U. De Sanctis, A. De Santo, J. B. De Vivie De Regie, W. J. Dearnaley, R. Debbe, C. Debenedetti, D. V. Dedovich, I. Deigaard, J. Del Peso, T. Del Prete, D. Delgove, F. Deliot, C. M. Delitzsch, M. Deliyergiyev, A. Dell’Acqua, L. Dell’Asta, M. Dell’Orso, M. Della Pietra, D. della Volpe, M. Delmastro, P. A. Delsart, C. Deluca, D. A. DeMarco, S. Demers, M. Demichev, A. Demilly, S. P. Denisov, D. Denysiuk, D. Derendarz, J. E. Derkaoui, F. Derue, P. Dervan, K. Desch, C. Deterre, K. Dette, P. O. Deviveiros, A. Dewhurst, S. Dhaliwal, A. Di Ciaccio, L. Di Ciaccio, W. K. Di Clemente, A. Di Domenico, C. Di Donato, A. Di Girolamo, B. Di Girolamo, A. Di Mattia, B. Di Micco, R. Di Nardo, A. Di Simone, R. Di Sipio, D. Di Valentino, C. Diaconu, M. Diamond, F. A. Dias, M. A. Diaz, E. B. Diehl, J. Dietrich, S. Diglio, A. Dimitrievska, J. Dingfelder, P. Dita, S. Dita, F. Dittus, F. Djama, T. Djobava, J. I. Djuvsland, M. A. B. do Vale, D. Dobos, M. Dobre, C. Doglioni, T. Dohmae, J. Dolejsi, Z. Dolezal, B. A. Dolgoshein, M. Donadelli, S. Donati, P. Dondero, J. Donini, J. Dopke, A. Doria, M. T. Dova, A. T. Doyle, E. Drechsler, M. Dris, Y. Du, J. Duarte-Campderros, E. Duchovni, G. Duckeck, O. A. Ducu, D. Duda, A. Dudarev, L. Duflot, L. Duguid, M. Dührssen, M. Dunford, H. Duran Yildiz, M. Düren, A. Durglishvili, D. Duschinger, B. Dutta, M. Dyndal, C. Eckardt, K. M. Ecker, R. C. Edgar, W. Edson, N. C. Edwards, T. Eifert, G. Eigen, K. Einsweiler, T. Ekelof, M. El Kacimi, V. Ellajosyula, M. Ellert, S. Elles, F. Ellinghaus, A. A. Elliot, N. Ellis, J. Elmsheuser, M. Elsing, D. Emeliyanov, Y. Enari, O. C. Endner, M. Endo, J. S. Ennis, J. Erdmann, A. Ereditato, G. Ernis, J. Ernst, M. Ernst, S. Errede, E. Ertel, M. Escalier, H. Esch, C. Escobar, B. Esposito, A. I. Etienvre, E. Etzion, H. Evans, A. Ezhilov, F. Fabbri, L. Fabbri, G. Facini, R. M. Fakhrutdinov, S. Falciano, R. J. Falla, J. Faltova, Y. Fang, M. Fanti, A. Farbin, A. Farilla, C. Farina, T. Farooque, S. Farrell, S. M. Farrington, P. Farthouat, F. Fassi, P. Fassnacht, D. Fassouliotis, M. Faucci Giannelli, A. Favareto, W. J. Fawcett, L. Fayard, O. L. Fedin, W. Fedorko, S. Feigl, L. Feligioni, C. Feng, E. J. Feng, H. Feng, A. B. Fenyuk, L. Feremenga, P. Fernandez Martinez, S. Fernandez Perez, J. Ferrando, A. Ferrari, P. Ferrari, R. Ferrari, D. E. Ferreira de Lima, A. Ferrer, D. Ferrere, C. Ferretti, A. Ferretto Parodi, F. Fiedler, A. Filipčič, M. Filipuzzi, F. Filthaut, M. Fincke-Keeler, K. D. Finelli, M. C. N. Fiolhais, L. Fiorini, A. Firan, A. Fischer, C. Fischer, J. Fischer, W. C. Fisher, N. Flaschel, I. Fleck, P. Fleischmann, G. T. Fletcher, G. Fletcher, R. R. M. Fletcher, T. Flick, A. Floderus, L. R. Flores Castillo, M. J. Flowerdew, G. T. Forcolin, A. Formica, A. Forti, A. G. Foster, D. Fournier, H. Fox, S. Fracchia, P. Francavilla, M. Franchini, D. Francis, L. Franconi, M. Franklin, M. Frate, M. Fraternali, D. Freeborn, S. M. Fressard-Batraneanu, F. Friedrich, D. Froidevaux, J. A. Frost, C. Fukunaga, E. Fullana Torregrosa, T. Fusayasu, J. Fuster, C. Gabaldon, O. Gabizon, A. Gabrielli, A. Gabrielli, G. P. Gach, S. Gadatsch, S. Gadomski, G. Gagliardi, L. G. Gagnon, P. Gagnon, C. Galea, B. Galhardo, E. J. Gallas, B. J. Gallop, P. Gallus, G. Galster, K. K. Gan, J. Gao, Y. Gao, Y. S. Gao, F. M. Garay Walls, C. García, J. E. García Navarro, M. Garcia-Sciveres, R. W. Gardner, N. Garelli, V. Garonne, A. Gascon Bravo, C. Gatti, A. Gaudiello, G. Gaudio, B. Gaur, L. Gauthier, I. L. Gavrilenko, C. Gay, G. Gaycken, E. N. Gazis, Z. Gecse, C. N. P. Gee, Ch. Geich-Gimbel, M. P. Geisler, C. Gemme, M. H. Genest, C. Geng, S. Gentile, S. George, D. Gerbaudo, A. Gershon, S. Ghasemi, H. Ghazlane, M. Ghneimat, B. Giacobbe, S. Giagu, P. Giannetti, B. Gibbard, S. M. Gibson, M. Gignac, M. Gilchriese, T. P. S. Gillam, D. Gillberg, G. Gilles, D. M. Gingrich, N. Giokaris, M. P. Giordani, F. M. Giorgi, F. M. Giorgi, P. F. Giraud, P. Giromini, D. Giugni, F. Giuli, C. Giuliani, M. Giulini, B. K. Gjelsten, S. Gkaitatzis, I. Gkialas, E. L. Gkougkousis, L. K. Gladilin, C. Glasman, J. Glatzer, P. C. F. Glaysher, A. Glazov, M. Goblirsch-Kolb, J. Godlewski, S. Goldfarb, T. Golling, D. Golubkov, A. Gomes, R. Gonçalo, J. Goncalves Pinto Firmino Da Costa, L. Gonella, A. Gongadze, S. González de la Hoz, G. Gonzalez Parra, S. Gonzalez-Sevilla, L. Goossens, P. A. Gorbounov, H. A. Gordon, I. Gorelov, B. Gorini, E. Gorini, A. Gorišek, E. Gornicki, A. T. Goshaw, C. Gössling, M. I. Gostkin, C. R. Goudet, D. Goujdami, A. G. Goussiou, N. Govender, E. Gozani, L. Graber, I. Grabowska-Bold, P. O. J. Gradin, P. Grafström, J. Gramling, E. Gramstad, S. Grancagnolo, V. Gratchev, H. M. Gray, E. Graziani, Z. D. Greenwood, C. Grefe, K. Gregersen, I. M. Gregor, P. Grenier, K. Grevtsov, J. Griffiths, A. A. Grillo, K. Grimm, S. Grinstein, Ph. Gris, J.-F. Grivaz, S. Groh, J. P. Grohs, E. Gross, J. Grosse-Knetter, G. C. Grossi, Z. J. Grout, L. Guan, W. Guan, J. Guenther, F. Guescini, D. Guest, O. Gueta, E. Guido, T. Guillemin, S. Guindon, U. Gul, C. Gumpert, J. Guo, Y. Guo, S. Gupta, G. Gustavino, P. Gutierrez, N. G. Gutierrez Ortiz, C. Gutschow, C. Guyot, C. Gwenlan, C. B. Gwilliam, A. Haas, C. Haber, H. K. Hadavand, N. Haddad, A. Hadef, P. Haefner, S. Hageböck, Z. Hajduk, H. Hakobyan, M. Haleem, J. Haley, D. Hall, G. Halladjian, G. D. Hallewell, K. Hamacher, P. Hamal, K. Hamano, A. Hamilton, G. N. Hamity, P. G. Hamnett, L. Han, K. Hanagaki, K. Hanawa, M. Hance, B. Haney, P. Hanke, R. Hanna, J. B. Hansen, J. D. Hansen, M. C. Hansen, P. H. Hansen, K. Hara, A. S. Hard, T. Harenberg, F. Hariri, S. Harkusha, R. D. Harrington, P. F. Harrison, F. Hartjes, M. Hasegawa, Y. Hasegawa, A. Hasib, S. Hassani, S. Haug, R. Hauser, L. Hauswald, M. Havranek, C. M. Hawkes, R. J. Hawkings, A. D. Hawkins, D. Hayden, C. P. Hays, J. M. Hays, H. S. Hayward, S. J. Haywood, S. J. Head, T. Heck, V. Hedberg, L. Heelan, S. Heim, T. Heim, B. Heinemann, J. J. Heinrich, L. Heinrich, C. Heinz, J. Hejbal, L. Helary, S. Hellman, C. Helsens, J. Henderson, R. C. W. Henderson, Y. Heng, S. Henkelmann, A. M. Henriques Correia, S. Henrot-Versille, G. H. Herbert, Y. Hernández Jiménez, G. Herten, R. Hertenberger, L. Hervas, G. G. Hesketh, N. P. Hessey, J. W. Hetherly, R. Hickling, E. Higón-Rodriguez, E. Hill, J. C. Hill, K. H. Hiller, S. J. Hillier, I. Hinchliffe, E. Hines, R. R. Hinman, M. Hirose, D. Hirschbuehl, J. Hobbs, N. Hod, M. C. Hodgkinson, P. Hodgson, A. Hoecker, M. R. Hoeferkamp, F. Hoenig, M. Hohlfeld, D. Hohn, T. R. Holmes, M. Homann, T. M. Hong, B. H. Hooberman, W. H. Hopkins, Y. Horii, A. J. Horton, J-Y. Hostachy, S. Hou, A. Hoummada, J. Howard, J. Howarth, M. Hrabovsky, I. Hristova, J. Hrivnac, T. Hryn’ova, A. Hrynevich, C. Hsu, P. J. Hsu, S.-C. Hsu, D. Hu, Q. Hu, Y. Huang, Z. Hubacek, F. Hubaut, F. Huegging, T. B. Huffman, E. W. Hughes, G. Hughes, M. Huhtinen, T. A. Hülsing, N. Huseynov, J. Huston, J. Huth, G. Iacobucci, G. Iakovidis, I. Ibragimov, L. Iconomidou-Fayard, E. Ideal, Z. Idrissi, P. Iengo, O. Igonkina, T. Iizawa, Y. Ikegami, M. Ikeno, Y. Ilchenko, D. Iliadis, N. Ilic, T. Ince, G. Introzzi, P. Ioannou, M. Iodice, K. Iordanidou, V. Ippolito, A. Irles Quiles, C. Isaksson, M. Ishino, M. Ishitsuka, R. Ishmukhametov, C. Issever, S. Istin, F. Ito, J. M. Iturbe Ponce, R. Iuppa, J. Ivarsson, W. Iwanski, H. Iwasaki, J. M. Izen, V. Izzo, S. Jabbar, B. Jackson, M. Jackson, P. Jackson, V. Jain, K. B. Jakobi, K. Jakobs, S. Jakobsen, T. Jakoubek, D. O. Jamin, D. K. Jana, E. Jansen, R. Jansky, J. Janssen, M. Janus, G. Jarlskog, N. Javadov, T. Javůrek, F. Jeanneau, L. Jeanty, J. Jejelava, G.-Y. Jeng, D. Jennens, P. Jenni, J. Jentzsch, C. Jeske, S. Jézéquel, H. Ji, J. Jia, H. Jiang, Y. Jiang, S. Jiggins, J. Jimenez Pena, S. Jin, A. Jinaru, O. Jinnouchi, P. Johansson, K. A. Johns, W. J. Johnson, K. Jon-And, G. Jones, R. W. L. Jones, S. Jones, T. J. Jones, J. Jongmanns, P. M. Jorge, J. Jovicevic, X. Ju, A. Juste Rozas, M. K. Köhler, A. Kaczmarska, M. Kado, H. Kagan, M. Kagan, S. J. Kahn, E. Kajomovitz, C. W. Kalderon, A. Kaluza, S. Kama, A. Kamenshchikov, N. Kanaya, S. Kaneti, V. A. Kantserov, J. Kanzaki, B. Kaplan, L. S. Kaplan, A. Kapliy, D. Kar, K. Karakostas, A. Karamaoun, N. Karastathis, M. J. Kareem, E. Karentzos, M. Karnevskiy, S. N. Karpov, Z. M. Karpova, K. Karthik, V. Kartvelishvili, A. N. Karyukhin, K. Kasahara, L. Kashif, R. D. Kass, A. Kastanas, Y. Kataoka, C. Kato, A. Katre, J. Katzy, K. Kawade, K. Kawagoe, T. Kawamoto, G. Kawamura, S. Kazama, V. F. Kazanin, R. Keeler, R. Kehoe, J. S. Keller, J. J. Kempster, H. Keoshkerian, O. Kepka, B. P. Kerševan, S. Kersten, R. A. Keyes, F. Khalil-zada, H. Khandanyan, A. Khanov, A. G. Kharlamov, T. J. Khoo, V. Khovanskiy, E. Khramov, J. Khubua, S. Kido, H. Y. Kim, S. H. Kim, Y. K. Kim, N. Kimura, O. M. Kind, B. T. King, M. King, S. B. King, J. Kirk, A. E. Kiryunin, T. Kishimoto, D. Kisielewska, F. Kiss, K. Kiuchi, O. Kivernyk, E. Kladiva, M. H. Klein, M. Klein, U. Klein, K. Kleinknecht, P. Klimek, A. Klimentov, R. Klingenberg, J. A. Klinger, T. Klioutchnikova, E.-E. Kluge, P. Kluit, S. Kluth, J. Knapik, E. Kneringer, E. B. F. G. Knoops, A. Knue, A. Kobayashi, D. Kobayashi, T. Kobayashi, M. Kobel, M. Kocian, P. Kodys, T. Koffas, E. Koffeman, L. A. Kogan, T. Kohriki, T. Koi, H. Kolanoski, M. Kolb, I. Koletsou, A. A. Komar, Y. Komori, T. Kondo, N. Kondrashova, K. Köneke, A. C. König, T. Kono, R. Konoplich, N. Konstantinidis, R. Kopeliansky, S. Koperny, L. Köpke, A. K. Kopp, K. Korcyl, K. Kordas, A. Korn, A. A. Korol, I. Korolkov, E. V. Korolkova, O. Kortner, S. Kortner, T. Kosek, V. V. Kostyukhin, V. M. Kotov, A. Kotwal, A. Kourkoumeli-Charalampidi, C. Kourkoumelis, V. Kouskoura, A. Koutsman, A. B. Kowalewska, R. Kowalewski, T. Z. Kowalski, W. Kozanecki, A. S. Kozhin, V. A. Kramarenko, G. Kramberger, D. Krasnopevtsev, M. W. Krasny, A. Krasznahorkay, J. K. Kraus, A. Kravchenko, M. Kretz, J. Kretzschmar, K. Kreutzfeldt, P. Krieger, K. Krizka, K. Kroeninger, H. Kroha, J. Kroll, J. Kroseberg, J. Krstic, U. Kruchonak, H. Krüger, N. Krumnack, A. Kruse, M. C. Kruse, M. Kruskal, T. Kubota, H. Kucuk, S. Kuday, J. T. Kuechler, S. Kuehn, A. Kugel, F. Kuger, A. Kuhl, T. Kuhl, V. Kukhtin, R. Kukla, Y. Kulchitsky, S. Kuleshov, M. Kuna, T. Kunigo, A. Kupco, H. Kurashige, Y. A. Kurochkin, V. Kus, E. S. Kuwertz, M. Kuze, J. Kvita, T. Kwan, D. Kyriazopoulos, A. La Rosa, J. L. La Rosa Navarro, L. La Rotonda, C. Lacasta, F. Lacava, J. Lacey, H. Lacker, D. Lacour, V. R. Lacuesta, E. Ladygin, R. Lafaye, B. Laforge, T. Lagouri, S. Lai, S. Lammers, W. Lampl, E. Lançon, U. Landgraf, M. P. J. Landon, V. S. Lang, J. C. Lange, A. J. Lankford, F. Lanni, K. Lantzsch, A. Lanza, S. Laplace, C. Lapoire, J. F. Laporte, T. Lari, F. Lasagni Manghi, M. Lassnig, P. Laurelli, W. Lavrijsen, A. T. Law, P. Laycock, T. Lazovich, M. Lazzaroni, O. Le Dortz, E. Le Guirriec, E. Le Menedeu, E. P. Le Quilleuc, M. LeBlanc, T. LeCompte, F. Ledroit-Guillon, C. A. Lee, S. C. Lee, L. Lee, G. Lefebvre, M. Lefebvre, F. Legger, C. Leggett, A. Lehan, G. Lehmann Miotto, X. Lei, W. A. Leight, A. Leisos, A. G. Leister, M. A. L. Leite, R. Leitner, D. Lellouch, B. Lemmer, K. J. C. Leney, T. Lenz, B. Lenzi, R. Leone, S. Leone, C. Leonidopoulos, S. Leontsinis, G. Lerner, C. Leroy, A. A. J. Lesage, C. G. Lester, M. Levchenko, J. Levêque, D. Levin, L. J. Levinson, M. Levy, A. M. Leyko, M. Leyton, B. Li, H. Li, H. L. Li, L. Li, L. Li, Q. Li, S. Li, X. Li, Y. Li, Z. Liang, H. Liao, B. Liberti, A. Liblong, P. Lichard, K. Lie, J. Liebal, W. Liebig, C. Limbach, A. Limosani, S. C. Lin, T. H. Lin, B. E. Lindquist, E. Lipeles, A. Lipniacka, M. Lisovyi, T. M. Liss, D. Lissauer, A. Lister, A. M. Litke, B. Liu, D. Liu, H. Liu, H. Liu, J. Liu, J. B. Liu, K. Liu, L. Liu, M. Liu, M. Liu, Y. L. Liu, Y. Liu, M. Livan, A. Lleres, J. Llorente Merino, S. L. Lloyd, F. Lo Sterzo, E. Lobodzinska, P. Loch, W. S. Lockman, F. K. Loebinger, A. E. Loevschall-Jensen, K. M. Loew, A. Loginov, T. Lohse, K. Lohwasser, M. Lokajicek, B. A. Long, J. D. Long, R. E. Long, L. Longo, K. A. Looper, L. Lopes, D. Lopez Mateos, B. Lopez Paredes, I. Lopez Paz, A. Lopez Solis, J. Lorenz, N. Lorenzo Martinez, M. Losada, P. J. Lösel, X. Lou, A. Lounis, J. Love, P. A. Love, H. Lu, N. Lu, H. J. Lubatti, C. Luci, A. Lucotte, C. Luedtke, F. Luehring, W. Lukas, L. Luminari, O. Lundberg, B. Lund-Jensen, D. Lynn, R. Lysak, E. Lytken, V. Lyubushkin, H. Ma, L. L. Ma, Y. Ma, G. Maccarrone, A. Macchiolo, C. M. Macdonald, B. Maček, J. Machado Miguens, D. Madaffari, R. Madar, H. J. Maddocks, W. F. Mader, A. Madsen, J. Maeda, S. Maeland, T. Maeno, A. Maevskiy, E. Magradze, J. Mahlstedt, C. Maiani, C. Maidantchik, A. A. Maier, T. Maier, A. Maio, S. Majewski, Y. Makida, N. Makovec, B. Malaescu, Pa. Malecki, V. P. Maleev, F. Malek, U. Mallik, D. Malon, C. Malone, S. Maltezos, V. M. Malyshev, S. Malyukov, J. Mamuzic, G. Mancini, B. Mandelli, L. Mandelli, I. Mandić, J. Maneira, L. Manhaes de Andrade Filho, J. Manjarres Ramos, A. Mann, B. Mansoulie, R. Mantifel, M. Mantoani, S. Manzoni, L. Mapelli, G. Marceca, L. March, G. Marchiori, M. Marcisovsky, M. Marjanovic, D. E. Marley, F. Marroquim, S. P. Marsden, Z. Marshall, L. F. Marti, S. Marti-Garcia, B. Martin, T. A. Martin, V. J. Martin, B. Martin dit Latour, M. Martinez, S. Martin-Haugh, V. S. Martoiu, A. C. Martyniuk, M. Marx, F. Marzano, A. Marzin, L. Masetti, T. Mashimo, R. Mashinistov, J. Masik, A. L. Maslennikov, I. Massa, L. Massa, P. Mastrandrea, A. Mastroberardino, T. Masubuchi, P. Mättig, J. Mattmann, J. Maurer, S. J. Maxfield, D. A. Maximov, R. Mazini, S. M. Mazza, N. C. Mc Fadden, G. Mc Goldrick, S. P. Mc Kee, A. McCarn, R. L. McCarthy, T. G. McCarthy, L. I. McClymont, K. W. McFarlane, J. A. Mcfayden, G. Mchedlidze, S. J. McMahon, R. A. McPherson, M. Medinnis, S. Meehan, S. Mehlhase, A. Mehta, K. Meier, C. Meineck, B. Meirose, B. R. Mellado Garcia, F. Meloni, A. Mengarelli, S. Menke, E. Meoni, K. M. Mercurio, S. Mergelmeyer, P. Mermod, L. Merola, C. Meroni, F. S. Merritt, A. Messina, J. Metcalfe, A. S. Mete, C. Meyer, C. Meyer, J.-P. Meyer, J. Meyer, H. Meyer Zu Theenhausen, R. P. Middleton, S. Miglioranzi, L. Mijović, G. Mikenberg, M. Mikestikova, M. Mikuž, M. Milesi, A. Milic, D. W. Miller, C. Mills, A. Milov, D. A. Milstead, A. A. Minaenko, Y. Minami, I. A. Minashvili, A. I. Mincer, B. Mindur, M. Mineev, Y. Ming, L. M. Mir, K. P. Mistry, T. Mitani, J. Mitrevski, V. A. Mitsou, A. Miucci, P. S. Miyagawa, J. U. Mjörnmark, T. Moa, K. Mochizuki, S. Mohapatra, W. Mohr, S. Molander, R. Moles-Valls, R. Monden, M. C. Mondragon, K. Mönig, J. Monk, E. Monnier, A. Montalbano, J. Montejo Berlingen, F. Monticelli, S. Monzani, R. W. Moore, N. Morange, D. Moreno, M. Moreno Llácer, P. Morettini, D. Mori, T. Mori, M. Morii, M. Morinaga, V. Morisbak, S. Moritz, A. K. Morley, G. Mornacchi, J. D. Morris, S. S. Mortensen, L. Morvaj, M. Mosidze, J. Moss, K. Motohashi, R. Mount, E. Mountricha, S. V. Mouraviev, E. J. W. Moyse, S. Muanza, R. D. Mudd, F. Mueller, J. Mueller, R. S. P. Mueller, T. Mueller, D. Muenstermann, P. Mullen, G. A. Mullier, F. J. Munoz Sanchez, J. A. Murillo Quijada, W. J. Murray, A. Murrone, H. Musheghyan, M. Muskinja, A. G. Myagkov, M. Myska, B. P. Nachman, O. Nackenhorst, J. Nadal, K. Nagai, R. Nagai, K. Nagano, Y. Nagasaka, K. Nagata, M. Nagel, E. Nagy, A. M. Nairz, Y. Nakahama, K. Nakamura, T. Nakamura, I. Nakano, H. Namasivayam, R. F. Naranjo Garcia, R. Narayan, D. I. Narrias Villar, I. Naryshkin, T. Naumann, G. Navarro, R. Nayyar, H. A. Neal, P. Yu. Nechaeva, T. J. Neep, P. D. Nef, A. Negri, M. Negrini, S. Nektarijevic, C. Nellist, A. Nelson, S. Nemecek, P. Nemethy, A. A. Nepomuceno, M. Nessi, M. S. Neubauer, M. Neumann, R. M. Neves, P. Nevski, P. R. Newman, D. H. Nguyen, R. B. Nickerson, R. Nicolaidou, B. Nicquevert, J. Nielsen, A. Nikiforov, V. Nikolaenko, I. Nikolic-Audit, K. Nikolopoulos, J. K. Nilsen, P. Nilsson, Y. Ninomiya, A. Nisati, R. Nisius, T. Nobe, L. Nodulman, M. Nomachi, I. Nomidis, T. Nooney, S. Norberg, M. Nordberg, N. Norjoharuddeen, O. Novgorodova, S. Nowak, M. Nozaki, L. Nozka, K. Ntekas, E. Nurse, F. Nuti, F. O’grady, D. C. O’Neil, A. A. O’Rourke, V. O’Shea, F. G. Oakham, H. Oberlack, T. Obermann, J. Ocariz, A. Ochi, I. Ochoa, J. P. Ochoa-Ricoux, S. Oda, S. Odaka, H. Ogren, A. Oh, S. H. Oh, C. C. Ohm, H. Ohman, H. Oide, H. Okawa, Y. Okumura, T. Okuyama, A. Olariu, L. F. Oleiro Seabra, S. A. Olivares Pino, D. Oliveira Damazio, A. Olszewski, J. Olszowska, A. Onofre, K. Onogi, P. U. E. Onyisi, C. J. Oram, M. J. Oreglia, Y. Oren, D. Orestano, N. Orlando, R. S. Orr, B. Osculati, R. Ospanov, G. Otero y Garzon, H. Otono, M. Ouchrif, F. Ould-Saada, A. Ouraou, K. P. Oussoren, Q. Ouyang, A. Ovcharova, M. Owen, R. E. Owen, V. E. Ozcan, N. Ozturk, K. Pachal, A. Pacheco Pages, C. Padilla Aranda, M. Pagáčová, S. Pagan Griso, F. Paige, P. Pais, K. Pajchel, G. Palacino, S. Palestini, M. Palka, D. Pallin, A. Palma, E. St. Panagiotopoulou, C. E. Pandini, J. G. Panduro Vazquez, P. Pani, S. Panitkin, D. Pantea, L. Paolozzi, Th. D. Papadopoulou, K. Papageorgiou, A. Paramonov, D. Paredes Hernandez, A. J. Parker, M. A. Parker, K. A. Parker, F. Parodi, J. A. Parsons, U. Parzefall, V. Pascuzzi, E. Pasqualucci, S. Passaggio, F. Pastore, Fr. Pastore, G. Pásztor, S. Pataraia, N. D. Patel, J. R. Pater, T. Pauly, J. Pearce, B. Pearson, L. E. Pedersen, M. Pedersen, S. Pedraza Lopez, R. Pedro, S. V. Peleganchuk, D. Pelikan, O. Penc, C. Peng, H. Peng, J. Penwell, B. S. Peralva, M. M. Perego, D. V. Perepelitsa, E. Perez Codina, L. Perini, H. Pernegger, S. Perrella, R. Peschke, V. D. Peshekhonov, K. Peters, R. F. Y. Peters, B. A. Petersen, T. C. Petersen, E. Petit, A. Petridis, C. Petridou, P. Petroff, E. Petrolo, M. Petrov, F. Petrucci, N. E. Pettersson, A. Peyaud, R. Pezoa, P. W. Phillips, G. Piacquadio, E. Pianori, A. Picazio, E. Piccaro, M. Piccinini, M. A. Pickering, R. Piegaia, J. E. Pilcher, A. D. Pilkington, A. W. J. Pin, J. Pina, M. Pinamonti, J. L. Pinfold, A. Pingel, S. Pires, H. Pirumov, M. Pitt, L. Plazak, M.-A. Pleier, V. Pleskot, E. Plotnikova, P. Plucinski, D. Pluth, R. Poettgen, L. Poggioli, D. Pohl, G. Polesello, A. Poley, A. Policicchio, R. Polifka, A. Polini, C. S. Pollard, V. Polychronakos, K. Pommès, L. Pontecorvo, B. G. Pope, G. A. Popeneciu, D. S. Popovic, A. Poppleton, S. Pospisil, K. Potamianos, I. N. Potrap, C. J. Potter, C. T. Potter, G. Poulard, J. Poveda, V. Pozdnyakov, M. E. Pozo Astigarraga, P. Pralavorio, A. Pranko, S. Prell, D. Price, L. E. Price, M. Primavera, S. Prince, M. Proissl, K. Prokofiev, F. Prokoshin, S. Protopopescu, J. Proudfoot, M. Przybycien, D. Puddu, D. Puldon, M. Purohit, P. Puzo, J. Qian, G. Qin, Y. Qin, A. Quadt, W. B. Quayle, M. Queitsch-Maitland, D. Quilty, S. Raddum, V. Radeka, V. Radescu, S. K. Radhakrishnan, P. Radloff, P. Rados, F. Ragusa, G. Rahal, J. A. Raine, S. Rajagopalan, M. Rammensee, C. Rangel-Smith, M. G. Ratti, F. Rauscher, S. Rave, T. Ravenscroft, M. Raymond, A. L. Read, N. P. Readioff, D. M. Rebuzzi, A. Redelbach, G. Redlinger, R. Reece, K. Reeves, L. Rehnisch, J. Reichert, H. Reisin, C. Rembser, H. Ren, M. Rescigno, S. Resconi, O. L. Rezanova, P. Reznicek, R. Rezvani, R. Richter, S. Richter, E. Richter-Was, O. Ricken, M. Ridel, P. Rieck, C. J. Riegel, J. Rieger, O. Rifki, M. Rijssenbeek, A. Rimoldi, L. Rinaldi, B. Ristić, E. Ritsch, I. Riu, F. Rizatdinova, E. Rizvi, C. Rizzi, S. H. Robertson, A. Robichaud-Veronneau, D. Robinson, J. E. M. Robinson, A. Robson, C. Roda, Y. Rodina, A. Rodriguez Perez, D. Rodriguez Rodriguez, S. Roe, C. S. Rogan, O. Røhne, A. Romaniouk, M. Romano, S. M. Romano Saez, E. Romero Adam, N. Rompotis, M. Ronzani, L. Roos, E. Ros, S. Rosati, K. Rosbach, P. Rose, O. Rosenthal, V. Rossetti, E. Rossi, L. P. Rossi, J. H. N. Rosten, R. Rosten, M. Rotaru, I. Roth, J. Rothberg, D. Rousseau, C. R. Royon, A. Rozanov, Y. Rozen, X. Ruan, F. Rubbo, I. Rubinskiy, V. I. Rud, M. S. Rudolph, F. Rühr, A. Ruiz-Martinez, Z. Rurikova, N. A. Rusakovich, A. Ruschke, H. L. Russell, J. P. Rutherfoord, N. Ruthmann, Y. F. Ryabov, M. Rybar, G. Rybkin, S. Ryu, A. Ryzhov, A. F. Saavedra, G. Sabato, S. Sacerdoti, H. F.-W. Sadrozinski, R. Sadykov, F. Safai Tehrani, P. Saha, M. Sahinsoy, M. Saimpert, T. Saito, H. Sakamoto, Y. Sakurai, G. Salamanna, A. Salamon, J. E. Salazar Loyola, D. Salek, P. H. Sales De Bruin, D. Salihagic, A. Salnikov, J. Salt, D. Salvatore, F. Salvatore, A. Salvucci, A. Salzburger, D. Sammel, D. Sampsonidis, A. Sanchez, J. Sánchez, V. Sanchez Martinez, H. Sandaker, R. L. Sandbach, H. G. Sander, M. P. Sanders, M. Sandhoff, C. Sandoval, R. Sandstroem, D. P. C. Sankey, M. Sannino, A. Sansoni, C. Santoni, R. Santonico, H. Santos, I. Santoyo Castillo, K. Sapp, A. Sapronov, J. G. Saraiva, B. Sarrazin, O. Sasaki, Y. Sasaki, K. Sato, G. Sauvage, E. Sauvan, G. Savage, P. Savard, C. Sawyer, L. Sawyer, J. Saxon, C. Sbarra, A. Sbrizzi, T. Scanlon, D. A. Scannicchio, M. Scarcella, V. Scarfone, J. Schaarschmidt, P. Schacht, D. Schaefer, R. Schaefer, J. Schaeffer, S. Schaepe, S. Schaetzel, U. Schäfer, A. C. Schaffer, D. Schaile, R. D. Schamberger, V. Scharf, V. A. Schegelsky, D. Scheirich, M. Schernau, C. Schiavi, C. Schillo, M. Schioppa, S. Schlenker, K. Schmieden, C. Schmitt, S. Schmitt, S. Schmitz, B. Schneider, Y. J. Schnellbach, U. Schnoor, L. Schoeffel, A. Schoening, B. D. Schoenrock, E. Schopf, A. L. S. Schorlemmer, M. Schott, J. Schovancova, S. Schramm, M. Schreyer, N. Schuh, M. J. Schultens, H.-C. Schultz-Coulon, H. Schulz, M. Schumacher, B. A. Schumm, Ph. Schune, C. Schwanenberger, A. Schwartzman, T. A. Schwarz, Ph. Schwegler, H. Schweiger, Ph. Schwemling, R. Schwienhorst, J. Schwindling, T. Schwindt, G. Sciolla, F. Scuri, F. Scutti, J. Searcy, P. Seema, S. C. Seidel, A. Seiden, F. Seifert, J. M. Seixas, G. Sekhniaidze, K. Sekhon, S. J. Sekula, D. M. Seliverstov, N. Semprini-Cesari, C. Serfon, L. Serin, L. Serkin, M. Sessa, R. Seuster, H. Severini, T. Sfiligoj, F. Sforza, A. Sfyrla, E. Shabalina, N. W. Shaikh, L. Y. Shan, R. Shang, J. T. Shank, M. Shapiro, P. B. Shatalov, K. Shaw, S. M. Shaw, A. Shcherbakova, C. Y. Shehu, P. Sherwood, L. Shi, S. Shimizu, C. O. Shimmin, M. Shimojima, M. Shiyakova, A. Shmeleva, D. Shoaleh Saadi, M. J. Shochet, S. Shojaii, S. Shrestha, E. Shulga, M. A. Shupe, P. Sicho, P. E. Sidebo, O. Sidiropoulou, D. Sidorov, A. Sidoti, F. Siegert, Dj. Sijacki, J. Silva, S. B. Silverstein, V. Simak, O. Simard, Lj. Simic, S. Simion, E. Simioni, B. Simmons, D. Simon, M. Simon, P. Sinervo, N. B. Sinev, M. Sioli, G. Siragusa, S. Yu. Sivoklokov, J. Sjölin, T. B. Sjursen, M. B. Skinner, H. P. Skottowe, P. Skubic, M. Slater, T. Slavicek, M. Slawinska, K. Sliwa, R. Slovak, V. Smakhtin, B. H. Smart, L. Smestad, S. Yu. Smirnov, Y. Smirnov, L. N. Smirnova, O. Smirnova, M. N. K. Smith, R. W. Smith, M. Smizanska, K. Smolek, A. A. Snesarev, G. Snidero, S. Snyder, R. Sobie, F. Socher, A. Soffer, D. A. Soh, G. Sokhrannyi, C. A. Solans Sanchez, M. Solar, E. Yu. Soldatov, U. Soldevila, A. A. Solodkov, A. Soloshenko, O. V. Solovyanov, V. Solovyev, P. Sommer, H. Son, H. Y. Song, A. Sood, A. Sopczak, V. Sopko, V. Sorin, D. Sosa, C. L. Sotiropoulou, R. Soualah, A. M. Soukharev, D. South, B. C. Sowden, S. Spagnolo, M. Spalla, M. Spangenberg, F. Spanò, D. Sperlich, F. Spettel, R. Spighi, G. Spigo, L. A. Spiller, M. Spousta, R. D. St. Denis, A. Stabile, S. Staerz, J. Stahlman, R. Stamen, S. Stamm, E. Stanecka, R. W. Stanek, C. Stanescu, M. Stanescu-Bellu, M. M. Stanitzki, S. Stapnes, E. A. Starchenko, G. H. Stark, J. Stark, P. Staroba, P. Starovoitov, R. Staszewski, P. Steinberg, B. Stelzer, H. J. Stelzer, O. Stelzer-Chilton, H. Stenzel, G. A. Stewart, J. A. Stillings, M. C. Stockton, M. Stoebe, G. Stoicea, P. Stolte, S. Stonjek, A. R. Stradling, A. Straessner, M. E. Stramaglia, J. Strandberg, S. Strandberg, A. Strandlie, M. Strauss, P. Strizenec, R. Ströhmer, D. M. Strom, R. Stroynowski, A. Strubig, S. A. Stucci, B. Stugu, N. A. Styles, D. Su, J. Su, R. Subramaniam, S. Suchek, Y. Sugaya, M. Suk, V. V. Sulin, S. Sultansoy, T. Sumida, S. Sun, X. Sun, J. E. Sundermann, K. Suruliz, G. Susinno, M. R. Sutton, S. Suzuki, M. Svatos, M. Swiatlowski, I. Sykora, T. Sykora, D. Ta, C. Taccini, K. Tackmann, J. Taenzer, A. Taffard, R. Tafirout, N. Taiblum, H. Takai, R. Takashima, H. Takeda, T. Takeshita, Y. Takubo, M. Talby, A. A. Talyshev, J. Y. C. Tam, K. G. Tan, J. Tanaka, R. Tanaka, S. Tanaka, B. B. Tannenwald, S. Tapia Araya, S. Tapprogge, S. Tarem, G. F. Tartarelli, P. Tas, M. Tasevsky, T. Tashiro, E. Tassi, A. Tavares Delgado, Y. Tayalati, A. C. Taylor, G. N. Taylor, P. T. E. Taylor, W. Taylor, F. A. Teischinger, P. Teixeira-Dias, K. K. Temming, D. Temple, H. Ten Kate, P. K. Teng, J. J. Teoh, F. Tepel, S. Terada, K. Terashi, J. Terron, S. Terzo, M. Testa, R. J. Teuscher, T. Theveneaux-Pelzer, J. P. Thomas, J. Thomas-Wilsker, E. N. Thompson, P. D. Thompson, R. J. Thompson, A. S. Thompson, L. A. Thomsen, E. Thomson, M. Thomson, M. J. Tibbetts, R. E. Ticse Torres, V. O. Tikhomirov, Yu. A. Tikhonov, S. Timoshenko, P. Tipton, S. Tisserant, K. Todome, T. Todorov, S. Todorova-Nova, J. Tojo, S. Tokár, K. Tokushuku, E. Tolley, L. Tomlinson, M. Tomoto, L. Tompkins, K. Toms, B. Tong, E. Torrence, H. Torres, E. Torró Pastor, J. Toth, F. Touchard, D. R. Tovey, T. Trefzger, L. Tremblet, A. Tricoli, I. M. Trigger, S. Trincaz-Duvoid, M. F. Tripiana, W. Trischuk, B. Trocmé, A. Trofymov, C. Troncon, M. Trottier-McDonald, M. Trovatelli, L. Truong, M. Trzebinski, A. Trzupek, J. C.-L. Tseng, P. V. Tsiareshka, G. Tsipolitis, N. Tsirintanis, S. Tsiskaridze, V. Tsiskaridze, E. G. Tskhadadze, K. M. Tsui, I. I. Tsukerman, V. Tsulaia, S. Tsuno, D. Tsybychev, A. Tudorache, V. Tudorache, A. N. Tuna, S. A. Tupputi, S. Turchikhin, D. Turecek, D. Turgeman, R. Turra, A. J. Turvey, P. M. Tuts, M. Tyndel, G. Ucchielli, I. Ueda, R. Ueno, M. Ughetto, F. Ukegawa, G. Unal, A. Undrus, G. Unel, F. C. Ungaro, Y. Unno, C. Unverdorben, J. Urban, P. Urquijo, P. Urrejola, G. Usai, A. Usanova, L. Vacavant, V. Vacek, B. Vachon, C. Valderanis, E. Valdes Santurio, N. Valencic, S. Valentinetti, A. Valero, L. Valery, S. Valkar, S. Vallecorsa, J. A. Valls Ferrer, W. Van Den Wollenberg, P. C. Van Der Deijl, R. van der Geer, H. van der Graaf, N. van Eldik, P. van Gemmeren, J. Van Nieuwkoop, I. van Vulpen, M. C. van Woerden, M. Vanadia, W. Vandelli, R. Vanguri, A. Vaniachine, P. Vankov, G. Vardanyan, R. Vari, E. W. Varnes, T. Varol, D. Varouchas, A. Vartapetian, K. E. Varvell, J. G. Vasquez, F. Vazeille, T. Vazquez Schroeder, J. Veatch, L. M. Veloce, F. Veloso, S. Veneziano, A. Ventura, M. Venturi, N. Venturi, A. Venturini, V. Vercesi, M. Verducci, W. Verkerke, J. C. Vermeulen, A. Vest, M. C. Vetterli, O. Viazlo, I. Vichou, T. Vickey, O. E. Vickey Boeriu, G. H. A. Viehhauser, S. Viel, L. Vigani, R. Vigne, M. Villa, M. Villaplana Perez, E. Vilucchi, M. G. Vincter, V. B. Vinogradov, C. Vittori, I. Vivarelli, S. Vlachos, M. Vlasak, M. Vogel, P. Vokac, G. Volpi, M. Volpi, H. von der Schmitt, E. von Toerne, V. Vorobel, K. Vorobev, M. Vos, R. Voss, J. H. Vossebeld, N. Vranjes, M. Vranjes Milosavljevic, V. Vrba, M. Vreeswijk, R. Vuillermet, I. Vukotic, Z. Vykydal, P. Wagner, W. Wagner, H. Wahlberg, S. Wahrmund, J. Wakabayashi, J. Walder, R. Walker, W. Walkowiak, V. Wallangen, C. Wang, C. Wang, F. Wang, H. Wang, H. Wang, J. Wang, J. Wang, K. Wang, R. Wang, S. M. Wang, T. Wang, T. Wang, X. Wang, C. Wanotayaroj, A. Warburton, C. P. Ward, D. R. Wardrope, A. Washbrook, P. M. Watkins, A. T. Watson, I. J. Watson, M. F. Watson, G. Watts, S. Watts, B. M. Waugh, S. Webb, M. S. Weber, S. W. Weber, J. S. Webster, A. R. Weidberg, B. Weinert, J. Weingarten, C. Weiser, H. Weits, P. S. Wells, T. Wenaus, T. Wengler, S. Wenig, N. Wermes, M. Werner, P. Werner, M. Wessels, J. Wetter, K. Whalen, N. L. Whallon, A. M. Wharton, A. White, M. J. White, R. White, S. White, D. Whiteson, F. J. Wickens, W. Wiedenmann, M. Wielers, P. Wienemann, C. Wiglesworth, L. A. M. Wiik-Fuchs, A. Wildauer, F. Wilk, H. G. Wilkens, H. H. Williams, S. Williams, C. Willis, S. Willocq, J. A. Wilson, I. Wingerter-Seez, F. Winklmeier, O. J. Winston, B. T. Winter, M. Wittgen, J. Wittkowski, S. J. Wollstadt, M. W. Wolter, H. Wolters, B. K. Wosiek, J. Wotschack, M. J. Woudstra, K. W. Wozniak, M. Wu, M. Wu, S. L. Wu, X. Wu, Y. Wu, T. R. Wyatt, B. M. Wynne, S. Xella, D. Xu, L. Xu, B. Yabsley, S. Yacoob, R. Yakabe, D. Yamaguchi, Y. Yamaguchi, A. Yamamoto, S. Yamamoto, T. Yamanaka, K. Yamauchi, Y. Yamazaki, Z. Yan, H. Yang, H. Yang, Y. Yang, Z. Yang, W-M. Yao, Y. C. Yap, Y. Yasu, E. Yatsenko, K. H. Yau Wong, J. Ye, S. Ye, I. Yeletskikh, A. L. Yen, E. Yildirim, K. Yorita, R. Yoshida, K. Yoshihara, C. Young, C. J. S. Young, S. Youssef, D. R. Yu, J. Yu, J. M. Yu, J. Yu, L. Yuan, S. P. Y. Yuen, I. Yusuff, B. Zabinski, R. Zaidan, A. M. Zaitsev, N. Zakharchuk, J. Zalieckas, A. Zaman, S. Zambito, L. Zanello, D. Zanzi, C. Zeitnitz, M. Zeman, A. Zemla, J. C. Zeng, Q. Zeng, K. Zengel, O. Zenin, T. Ženiš, D. Zerwas, D. Zhang, F. Zhang, G. Zhang, H. Zhang, J. Zhang, L. Zhang, R. Zhang, R. Zhang, X. Zhang, Z. Zhang, X. Zhao, Y. Zhao, Z. Zhao, A. Zhemchugov, J. Zhong, B. Zhou, C. Zhou, L. Zhou, L. Zhou, M. Zhou, N. Zhou, C. G. Zhu, H. Zhu, J. Zhu, Y. Zhu, X. Zhuang, K. Zhukov, A. Zibell, D. Zieminska, N. I. Zimine, C. Zimmermann, S. Zimmermann, Z. Zinonos, M. Zinser, M. Ziolkowski, L. Živković, G. Zobernig, A. Zoccoli, M. zur Nedden, G. Zurzolo, L. Zwalinski, ATLAS Collaboration

**Affiliations:** 1 Department of Physics, University of Adelaide, Adelaide, Australia; 2 Physics Department, SUNY Albany, Albany, NY USA; 3 Department of Physics, University of Alberta, Edmonton, AB Canada; 4 Department of Physics, Ankara University, Ankara, Turkey; 5 Istanbul Aydin University, Istanbul, Turkey; 6 Division of Physics, TOBB University of Economics and Technology, Ankara, Turkey; 7 LAPP, CNRS/IN2P3 and Université Savoie Mont Blanc, Annecy-le-Vieux, France; 8 High Energy Physics Division, Argonne National Laboratory, Argonne, IL USA; 9 Department of Physics, University of Arizona, Tucson, AZ USA; 10 Department of Physics, The University of Texas at Arlington, Arlington, TX USA; 11 Physics Department, University of Athens, Athens, Greece; 12 Physics Department, National Technical University of Athens, Zografou, Greece; 13 Institute of Physics, Azerbaijan Academy of Sciences, Baku, Azerbaijan; 14 Institut de Física d’Altes Energies (IFAE), The Barcelona Institute of Science and Technology, Barcelona, Spain; 15 Institute of Physics, University of Belgrade, Belgrade, Serbia; 16 Department for Physics and Technology, University of Bergen, Bergen, Norway; 17 Physics Division, Lawrence Berkeley National Laboratory and University of California, Berkeley, CA USA; 18 Department of Physics, Humboldt University, Berlin, Germany; 19 Albert Einstein Center for Fundamental Physics and Laboratory for High Energy Physics, University of Bern, Bern, Switzerland; 20 School of Physics and Astronomy, University of Birmingham, Birmingham, UK; 21 Department of Physics, Bogazici University, Istanbul, Turkey; 22 Department of Physics Engineering, Gaziantep University, Gaziantep, Turkey; 23 Faculty of Engineering and Natural Sciences, Istanbul Bilgi University, Istanbul, Turkey; 24 Faculty of Engineering and Natural Sciences, Bahcesehir University, Istanbul, Turkey; 25 Centro de Investigaciones, Universidad Antonio Narino, Bogotá, Colombia; 26 INFN Sezione di Bologna, Bologna, Italy; 27 Dipartimento di Fisica e Astronomia, Università di Bologna, Bologna, Italy; 28 Physikalisches Institut, University of Bonn, Bonn, Germany; 29 Department of Physics, Boston University, Boston, MA USA; 30 Department of Physics, Brandeis University, Waltham, MA USA; 31 Universidade Federal do Rio De Janeiro COPPE/EE/IF, Rio de Janeiro, Brazil; 32 Electrical Circuits Department, Federal University of Juiz de Fora (UFJF), Juiz de Fora, Brazil; 33 Federal University of Sao Joao del Rei (UFSJ), São João del Rei, Brazil; 34 Instituto de Fisica, Universidade de Sao Paulo, São Paulo, Brazil; 35 Physics Department, Brookhaven National Laboratory, Upton, NY USA; 36 Transilvania University of Brasov, Brasov, Romania; 37 National Institute of Physics and Nuclear Engineering, Bucharest, Romania; 38 Physics Department, National Institute for Research and Development of Isotopic and Molecular Technologies, Cluj Napoca, Romania; 39 University Politehnica Bucharest, Bucharest, Romania; 40 West University in Timisoara, Timisoara, Romania; 41 Departamento de Física, Universidad de Buenos Aires, Buenos Aires, Argentina; 42 Cavendish Laboratory, University of Cambridge, Cambridge, UK; 43 Department of Physics, Carleton University, Ottawa, ON Canada; 44 CERN, Geneva, Switzerland; 45 Enrico Fermi Institute, University of Chicago, Chicago, IL USA; 46 Departamento de Física, Pontificia Universidad Católica de Chile, Santiago, Chile; 47 Departamento de Física, Universidad Técnica Federico Santa María, Valparaiso, Chile; 48 Institute of High Energy Physics, Chinese Academy of Sciences, Beijing, China; 49 Department of Modern Physics, University of Science and Technology of China, Hefei, Anhui China; 50 Department of Physics, Nanjing University, Nanjing, Jiangsu China; 51 School of Physics, Shandong University, Jinan, Shandong China; 52 Shanghai Key Laboratory for Particle Physics and Cosmology, Department of Physics and Astronomy, Shanghai Jiao Tong University (also affiliated with PKU-CHEP), Shanghai, China; 53 Physics Department, Tsinghua University, Beijing, 100084 China; 54 Laboratoire de Physique Corpusculaire, Clermont Université and Université Blaise Pascal and CNRS/IN2P3, Clermont-Ferrand, France; 55 Nevis Laboratory, Columbia University, Irvington, NY USA; 56 Niels Bohr Institute, University of Copenhagen, Kobenhavn, Denmark; 57 INFN Gruppo Collegato di Cosenza, Laboratori Nazionali di Frascati, Frascati, Italy; 58 Dipartimento di Fisica, Università della Calabria, Rende, Italy; 59 Faculty of Physics and Applied Computer Science, AGH University of Science and Technology, Kraków, Poland; 60 Marian Smoluchowski Institute of Physics, Jagiellonian University, Kraków, Poland; 61 Institute of Nuclear Physics, Polish Academy of Sciences, Kraków, Poland; 62 Physics Department, Southern Methodist University, Dallas, TX USA; 63 Physics Department, University of Texas at Dallas, Richardson, TX USA; 64 DESY, Hamburg and Zeuthen, Germany; 65 Institut für Experimentelle Physik IV, Technische Universität Dortmund, Dortmund, Germany; 66 Institut für Kern- und Teilchenphysik, Technische Universität Dresden, Dresden, Germany; 67 Department of Physics, Duke University, Durham, NC USA; 68 SUPA-School of Physics and Astronomy, University of Edinburgh, Edinburgh, UK; 69 INFN Laboratori Nazionali di Frascati, Frascati, Italy; 70 Fakultät für Mathematik und Physik, Albert-Ludwigs-Universität, Freiburg, Germany; 71 Section de Physique, Université de Genève, Geneva, Switzerland; 72 INFN Sezione di Genova, Genoa, Italy; 73 Dipartimento di Fisica, Università di Genova, Genoa, Italy; 74 E. Andronikashvili Institute of Physics, Iv. Javakhishvili Tbilisi State University, Tbilisi, Georgia; 75 High Energy Physics Institute, Tbilisi State University, Tbilisi, Georgia; 76 II Physikalisches Institut, Justus-Liebig-Universität Giessen, Giessen, Germany; 77 SUPA-School of Physics and Astronomy, University of Glasgow, Glasgow, UK; 78 II Physikalisches Institut, Georg-August-Universität, Göttingen, Germany; 79 Laboratoire de Physique Subatomique et de Cosmologie, Université Grenoble-Alpes, CNRS/IN2P3, Grenoble, France; 80 Department of Physics, Hampton University, Hampton VA, USA; 81 Laboratory for Particle Physics and Cosmology, Harvard University, Cambridge, MA USA; 82 Kirchhoff-Institut für Physik, Ruprecht-Karls-Universität Heidelberg, Heidelberg, Germany; 83 Physikalisches Institut, Ruprecht-Karls-Universität Heidelberg, Heidelberg, Germany; 84 ZITI Institut für technische Informatik, Ruprecht-Karls-Universität Heidelberg, Mannheim, Germany; 85 Faculty of Applied Information Science, Hiroshima Institute of Technology, Hiroshima, Japan; 86 Department of Physics, The Chinese University of Hong Kong, Shatin, NT Hong Kong; 87 Department of Physics, The University of Hong Kong, Hong Kong, China; 88 Department of Physics, The Hong Kong University of Science and Technology, Clear Water Bay, Kowloon, Hong Kong, China; 89 Department of Physics, Indiana University, Bloomington, IN USA; 90 Institut für Astro- und Teilchenphysik, Leopold-Franzens-Universität, Innsbruck, Austria; 91 University of Iowa, Iowa City, IA USA; 92 Department of Physics and Astronomy, Iowa State University, Ames, IA USA; 93 Department of Physics and Astronomy, University of California Irvine, Irvine, CA USA; 94 Joint Institute for Nuclear Research, JINR Dubna, Dubna, Russia; 95 KEK, High Energy Accelerator Research Organization, Tsukuba, Japan; 96 Graduate School of Science, Kobe University, Kobe, Japan; 97 Faculty of Science, Kyoto University, Kyoto, Japan; 98 Kyoto University of Education, Kyoto, Japan; 99 Department of Physics, Kyushu University, Fukuoka, Japan; 100 Instituto de Física La Plata, Universidad Nacional de La Plata and CONICET, La Plata, Argentina; 101 Physics Department, Lancaster University, Lancaster, UK; 102 INFN Sezione di Lecce, Lecce, Italy; 103 Dipartimento di Matematica e Fisica, Università del Salento, Lecce, Italy; 104 Oliver Lodge Laboratory, University of Liverpool, Liverpool, UK; 105 Department of Physics, Jožef Stefan Institute and University of Ljubljana, Ljubljana, Slovenia; 106 School of Physics and Astronomy, Queen Mary University of London, London, UK; 107 Department of Physics, Royal Holloway University of London, Surrey, UK; 108 Department of Physics and Astronomy, University College London, London, UK; 109 Louisiana Tech University, Ruston, LA USA; 110 Laboratoire de Physique Nucléaire et de Hautes Energies, UPMC and Université Paris-Diderot and CNRS/IN2P3, Paris, France; 111 Fysiska institutionen, Lunds universitet, Lund, Sweden; 112 Departamento de Fisica Teorica C-15, Universidad Autonoma de Madrid, Madrid, Spain; 113 Institut für Physik, Universität Mainz, Mainz, Germany; 114 School of Physics and Astronomy, University of Manchester, Manchester, UK; 115 CPPM, Aix-Marseille Université and CNRS/IN2P3, Marseille, France; 116 Department of Physics, University of Massachusetts, Amherst, MA USA; 117 Department of Physics, McGill University, Montreal, QC Canada; 118 School of Physics, University of Melbourne, Melbourne, VIC Australia; 119 Department of Physics, The University of Michigan, Ann Arbor, MI USA; 120 Department of Physics and Astronomy, Michigan State University, East Lansing, MI USA; 121 INFN Sezione di Milano, Milan, Italy; 122 Dipartimento di Fisica, Università di Milano, Milan, Italy; 123 B.I. Stepanov Institute of Physics, National Academy of Sciences of Belarus, Minsk, Republic of Belarus; 124 National Scientific and Educational Centre for Particle and High Energy Physics, Minsk, Republic of Belarus; 125 Group of Particle Physics, University of Montreal, Montreal, QC Canada; 126 P.N. Lebedev Physical Institute of the Russian Academy of Sciences, Moscow, Russia; 127 Institute for Theoretical and Experimental Physics (ITEP), Moscow, Russia; 128 National Research Nuclear University MEPhI, Moscow, Russia; 129 D.V. Skobeltsyn Institute of Nuclear Physics, M.V. Lomonosov Moscow State University, Moscow, Russia; 130 Fakultät für Physik, Ludwig-Maximilians-Universität München, Munich, Germany; 131 Max-Planck-Institut für Physik (Werner-Heisenberg-Institut), Munich, Germany; 132 Nagasaki Institute of Applied Science, Nagasaki, Japan; 133 Graduate School of Science and Kobayashi-Maskawa Institute, Nagoya University, Nagoya, Japan; 134 INFN Sezione di Napoli, Naples, Italy; 135 Dipartimento di Fisica, Università di Napoli, Naples, Italy; 136 Department of Physics and Astronomy, University of New Mexico, Albuquerque, NM USA; 137 Institute for Mathematics, Astrophysics and Particle Physics, Radboud University Nijmegen/Nikhef, Nijmegen, The Netherlands; 138 Nikhef National Institute for Subatomic Physics and University of Amsterdam, Amsterdam, The Netherlands; 139 Department of Physics, Northern Illinois University, DeKalb, IL USA; 140 Budker Institute of Nuclear Physics, SB RAS, Novosibirsk, Russia; 141 Department of Physics, New York University, New York, NY USA; 142 Ohio State University, Columbus, OH USA; 143 Faculty of Science, Okayama University, Okayama, Japan; 144 Homer L. Dodge Department of Physics and Astronomy, University of Oklahoma, Norman, OK USA; 145 Department of Physics, Oklahoma State University, Stillwater, OK USA; 146 Palacký University, RCPTM, Olomouc, Czech Republic; 147 Center for High Energy Physics, University of Oregon, Eugene, OR USA; 148 LAL, University of Paris-Sud, CNRS/IN2P3, Université Paris-Saclay, Orsay, France; 149 Graduate School of Science, Osaka University, Osaka, Japan; 150 Department of Physics, University of Oslo, Oslo, Norway; 151 Department of Physics, Oxford University, Oxford, UK; 152 INFN Sezione di Pavia, Pavia, Italy; 153 Dipartimento di Fisica, Università di Pavia, Pavia, Italy; 154 Department of Physics, University of Pennsylvania, Philadelphia, PA USA; 155 National Research Centre “Kurchatov Institute” B.P. Konstantinov Petersburg Nuclear Physics Institute, St. Petersburg, Russia; 156 INFN Sezione di Pisa, Pisa, Italy; 157 Dipartimento di Fisica E. Fermi, Università di Pisa, Pisa, Italy; 158 Department of Physics and Astronomy, University of Pittsburgh, Pittsburgh, PA USA; 159 Laboratório de Instrumentação e Física Experimental de Partículas-LIP, Lisbon, Portugal; 160 Faculdade de Ciências, Universidade de Lisboa, Lisbon, Portugal; 161 Department of Physics, University of Coimbra, Coimbra, Portugal; 162 Centro de Física Nuclear da Universidade de Lisboa, Lisbon, Portugal; 163 Departamento de Fisica, Universidade do Minho, Braga, Portugal; 164 Departamento de Fisica Teorica y del Cosmos and CAFPE, Universidad de Granada, Granada, Spain; 165 Dep Fisica and CEFITEC of Faculdade de Ciencias e Tecnologia, Universidade Nova de Lisboa, Caparica, Portugal; 166 Institute of Physics, Academy of Sciences of the Czech Republic, Prague, Czech Republic; 167 Czech Technical University in Prague, Prague, Czech Republic; 168 Faculty of Mathematics and Physics, Charles University in Prague, Prague, Czech Republic; 169 State Research Center Institute for High Energy Physics (Protvino), NRC KI, Protvino, Russia; 170 Particle Physics Department, Rutherford Appleton Laboratory, Didcot, UK; 171 INFN Sezione di Roma, Rome, Italy; 172 Dipartimento di Fisica, Sapienza Università di Roma, Rome, Italy; 173 INFN Sezione di Roma Tor Vergata, Rome, Italy; 174 Dipartimento di Fisica, Università di Roma Tor Vergata, Rome, Italy; 175 INFN Sezione di Roma Tre, Rome, Italy; 176 Dipartimento di Matematica e Fisica, Università Roma Tre, Rome, Italy; 177 Faculté des Sciences Ain Chock, Réseau Universitaire de Physique des Hautes Energies-Université Hassan II, Casablanca, Morocco; 178 Centre National de l’Energie des Sciences Techniques Nucleaires, Rabat, Morocco; 179 Faculté des Sciences Semlalia, Université Cadi Ayyad, LPHEA-Marrakech, Marrakech, Morocco; 180 Faculté des Sciences, Université Mohamed Premier and LPTPM, Oujda, Morocco; 181 Faculté des Sciences, Université Mohammed V, Rabat, Morocco; 182 DSM/IRFU (Institut de Recherches sur les Lois Fondamentales de l’Univers), CEA Saclay (Commissariat à l’Energie Atomique et aux Energies Alternatives), Gif-sur-Yvette, France; 183 Santa Cruz Institute for Particle Physics, University of California Santa Cruz, Santa Cruz, CA USA; 184 Department of Physics, University of Washington, Seattle, WA USA; 185 Department of Physics and Astronomy, University of Sheffield, Sheffield, UK; 186 Department of Physics, Shinshu University, Nagano, Japan; 187 Fachbereich Physik, Universität Siegen, Siegen, Germany; 188 Department of Physics, Simon Fraser University, Burnaby, BC Canada; 189 SLAC National Accelerator Laboratory, Stanford, CA USA; 190 Faculty of Mathematics, Physics and Informatics, Comenius University, Bratislava, Slovak Republic; 191 Department of Subnuclear Physics, Institute of Experimental Physics of the Slovak Academy of Sciences, Kosice, Slovak Republic; 192 Department of Physics, University of Cape Town, Cape Town, South Africa; 193 Department of Physics, University of Johannesburg, Johannesburg, South Africa; 194 School of Physics, University of the Witwatersrand, Johannesburg, South Africa; 195 Department of Physics, Stockholm University, Stockholm, Sweden; 196 The Oskar Klein Centre, Stockholm, Sweden; 197 Physics Department, Royal Institute of Technology, Stockholm, Sweden; 198 Departments of Physics and Astronomy and Chemistry, Stony Brook University, Stony Brook, NY USA; 199 Department of Physics and Astronomy, University of Sussex, Brighton, UK; 200 School of Physics, University of Sydney, Sydney, Australia; 201 Institute of Physics, Academia Sinica, Taipei, Taiwan; 202 Department of Physics, Technion: Israel Institute of Technology, Haifa, Israel; 203 Raymond and Beverly Sackler School of Physics and Astronomy, Tel Aviv University, Tel Aviv, Israel; 204 Department of Physics, Aristotle University of Thessaloniki, Thessaloniki, Greece; 205 International Center for Elementary Particle Physics and Department of Physics, The University of Tokyo, Tokyo, Japan; 206 Graduate School of Science and Technology, Tokyo Metropolitan University, Tokyo, Japan; 207 Department of Physics, Tokyo Institute of Technology, Tokyo, Japan; 208 Department of Physics, University of Toronto, Toronto, ON Canada; 209 TRIUMF, Vancouver, BC Canada; 210 Department of Physics and Astronomy, York University, Toronto, ON Canada; 211 Faculty of Pure and Applied Sciences, and Center for Integrated Research in Fundamental Science and Engineering, University of Tsukuba, Tsukuba, Japan; 212 Department of Physics and Astronomy, Tufts University, Medford, MA USA; 213 INFN Gruppo Collegato di Udine, Sezione di Trieste, Udine, Italy; 214 ICTP, Trieste, Italy; 215 Dipartimento di Chimica Fisica e Ambiente, Università di Udine, Udine, Italy; 216 Department of Physics and Astronomy, University of Uppsala, Uppsala, Sweden; 217 Department of Physics, University of Illinois, Urbana, IL USA; 218 Instituto de Física Corpuscular (IFIC) and Departamento de Física Atómica, Molecular y Nuclear and Departamento de Ingeniería Electrónica and Instituto de Microelectrónica de Barcelona (IMB-CNM), University of Valencia and CSIC, Valencia, Spain; 219 Department of Physics, University of British Columbia, Vancouver, BC Canada; 220 Department of Physics and Astronomy, University of Victoria, Victoria, BC Canada; 221 Department of Physics, University of Warwick, Coventry, UK; 222 Waseda University, Tokyo, Japan; 223 Department of Particle Physics, The Weizmann Institute of Science, Rehovot, Israel; 224 Department of Physics, University of Wisconsin, Madison, WI USA; 225 Fakultät für Physik und Astronomie, Julius-Maximilians-Universität, Würzburg, Germany; 226 Fakultät für Mathematik und Naturwissenschaften, Fachgruppe Physik, Bergische Universität Wuppertal, Wuppertal, Germany; 227 Department of Physics, Yale University, New Haven, CT USA; 228 Yerevan Physics Institute, Yerevan, Armenia; 229 Centre de Calcul de l’Institut National de Physique Nucléaire et de Physique des Particules (IN2P3), Villeurbanne, France; 230 CERN, 1211 Geneva 23, Switzerland

## Abstract

A test of CP invariance in Higgs boson production via vector-boson fusion using the method of the *Optimal Observable* is presented. The analysis exploits the decay mode of the Higgs boson into a pair of $\tau$ leptons and is based on 20.3 $\mathrm{fb}^{-1}$ of proton–proton collision data at $\sqrt{s}$ = 8 $\,\mathrm{TeV}$ collected by the ATLAS experiment at the LHC. Contributions from CP-violating interactions between the Higgs boson and electroweak gauge bosons are described in an effective field theory framework, in which the strength of CP violation is governed by a single parameter $\tilde{d}$. The mean values and distributions of CP-odd observables agree with the expectation in the Standard Model and show no sign of CP violation. The CP-mixing parameter $\tilde{d}$ is constrained to the interval $(-0.11,0.05)$ at 68% confidence level, consistent with the Standard Model expectation of $\tilde{d}=0$.

## Introduction

The discovery of a Higgs boson by the ATLAS and CMS experiments [1, 2] at the LHC [3] offers a novel opportunity to search for new sources of CP violation in the interaction of the Higgs boson with other Standard Model (SM) particles. C and CP violation is one of the three Sakharov conditions [4–6] needed to explain the observed baryon asymmetry of the universe. In the SM with massless neutrinos the only source of CP violation is the complex phase in the quark mixing (CKM) matrix [7, 8]. The measured size of the complex phase and the derived magnitude of CP violation in the early universe is insufficient to explain the observed value of the baryon asymmetry [9] within the SM [10, 11] and, most probably, new sources of CP violation beyond the SM need to be introduced. No observable effect of CP violation is expected in the production or decay of the SM Higgs boson. Hence any observation of CP violation involving the observed Higgs boson would be an unequivocal sign of physics beyond the SM.

The measured Higgs boson production cross sections, branching ratios and derived constraints on coupling-strength modifiers, assuming the tensor structure of the SM, agree with the SM predictions [12, 13]. Investigations of spin and CP quantum numbers in bosonic decay modes and measurements of anomalous couplings including CP-violating ones in the decay into a pair of massive electroweak gauge bosons show no hints of deviations from the tensor structure of the SM Higgs boson [14, 15]. Differential cross-section measurements in the decay $H \rightarrow \gamma \gamma$ have been used to set limits on couplings including CP-violating ones in vector-boson fusion production in an effective field theory [16]. However, the observables, including absolute event rates, used in that analysis were CP-even and hence not sensitive to the possible interference between the SM and CP-odd couplings and did not directly test CP invariance. The observables used in this analysis are CP-odd and therefore sensitive to this interference and the measurement is designed as a direct test of CP invariance.

In this paper, a first direct test of CP invariance in Higgs boson production via vector-boson fusion (VBF) is presented, based on proton–proton collision data corresponding to an integrated luminosity of 20.3 fb$^{-1}$  collected with the ATLAS detector at $\sqrt{s}$ = 8 $\,\mathrm{TeV}$ in 2012. A CP-odd *Optimal Observable* [17–19] is employed. The *Optimal Observable* combines the information from the multi-dimensional phase space in a single quantity calculated from leading-order matrix elements for VBF production. Hence it does not depend on the decay mode of the Higgs boson. A direct test of CP invariance is possible measuring the mean value of the CP-odd *Optimal Observable*. Moreover, as described in Sect. 2, an ansatz in the framework of an effective field theory is utilised, in which all CP-violating effects corresponding to operators with dimensions up to six in the couplings between a Higgs boson and an electroweak gauge boson can be described in terms of a single parameter $\tilde{d}$. Limits on $\tilde{d}$ are derived by analysing the shape of spectra of the *Optimal Observable* measured in $H\rightarrow \tau \tau$ candidate events that also have two jets tagging VBF production. The event selection, estimation of background contributions and of systematic uncertainties follows the analysis used to establish $4.5\sigma$ evidence for the $H\rightarrow \tau \tau$ decay [20]. Only events selected in the VBF category are analysed, and only fully leptonic $\tau _{\mathrm {lep}}\tau _{\mathrm {lep}}$ or semileptonic $\tau _{\mathrm {lep}}\tau _{\mathrm {had}}$ decays of the $\tau$-lepton pair are considered.

The theoretical framework in the context of effective field theories is discussed in Sect. 2 and the methodology of testing CP invariance and the concept of the *Optimal Observable* are introduced in Sect. 3. After a brief description of the ATLAS detector in Sect. 4, the simulated samples used are summarised in Sect. 5. The experimental analysis is presented in Sect. 6, followed by a description of the statistical method used to determine confidence intervals for $\tilde{d}$ in Sect. 7. The results are discussed in Sect. 8, following which conclusions are given.

## Effective Lagrangian framework

The effective Lagrangian considered is the SM Lagrangian augmented by CP-violating operators of mass dimension six, which can be constructed from the Higgs doublet $\Phi$ and the U(1)$_Y$ and SU(2)$_{I_W,\mathrm{L}}$ electroweak gauge fields $B^\mu$ and $W^{a,\mu }$ $(a = 1,2,3)$, respectively. No CP-conserving dimension-six operators built from these fields are taken into account. All interactions between the Higgs boson and other SM particles (fermions and gluons) are assumed to be as predicted in the SM; i.e. the coupling structure in gluon fusion production and in the decay into a pair of $\tau$-leptons is considered to be the same as in the SM.

The effective U(1)$_Y$- and SU(2)$_{I_W,\mathrm{L}}$-invariant Lagrangian is then given by (following Ref. [21, 22]):

1 $$\begin{aligned} \mathcal{L}_{\mathrm {eff}} = \mathcal{L}_{\mathrm {SM}} + \frac{f_{\tilde{B}B}}{\Lambda ^2}\mathcal{O}_{\tilde{B}B} + \frac{f_{\tilde{W}W}}{\Lambda ^2}\mathcal{O}_{\tilde{W}W} + \frac{f_{\tilde{B}}}{\Lambda ^2}\mathcal{O}_{\tilde{B}} \end{aligned}$$

with the three dimension-six operators

2 $$\begin{aligned}&\mathcal{O}_{\tilde{B}B} = \Phi^+ \hat{\tilde{B}}_{\mu\nu}\hat{B}^{\mu\nu} \Phi \nonumber\\&\mathcal{O}_{\tilde{W}W} = \Phi^+\hat{\tilde{W}}_{\mu\nu} \hat{W}^{\mu\nu} \Phi\nonumber\\&\mathcal{O}_{\tilde{B}} = (D_\mu \Phi)^+ \hat{\tilde{B}}^{\mu\nu}D_\nu \Phi.\end{aligned}$$

and three dimensionless Wilson coefficients $f_{\tilde{B}B}$, $f_{\tilde{W}W}$ and $f_{\tilde{B}}$; $\Lambda$ is the scale of new physics.

Here $D_\mu$ denotes the covariant derivative $D_\mu = \partial _\mu +\frac{\mathrm {i}}{2}g^\prime B_\mu + \mathrm {i} g \frac{\sigma _a}{2} W_\mu ^a$, $\hat{V}_{\mu \nu }$ ($V = B, W^a$) the field-strength tensors and $\tilde{V}_{\mu \nu } = \frac{1}{2} \epsilon _{\mu \nu \rho \sigma }{V}^{\rho \sigma }$ the dual field-strength tensors, with $\hat{B}_{\mu \nu } + \hat{W}_{\mu \nu } = \mathrm {i} \frac{g^\prime }{2} B_{\mu \nu } + \mathrm {i} \frac{g}{2} \sigma ^a W^a_{\mu \nu }$.

The last operator $\mathcal{O}_{\tilde{B}}$ contributes to the CP-violating charged triple gauge-boson couplings $\tilde{\kappa }_{\gamma }$ and $\tilde{\kappa }_{Z}$ via the relation $\tilde{\kappa }_{\gamma } = - \cot ^2 \theta _W \tilde{\kappa }_{Z} = \frac{m_W^2}{2\Lambda ^2} f_{\tilde{B}}$. These CP-violating charged triple gauge boson couplings are constrained by the LEP experiments [23–25] and the contribution from $\mathcal{O}_{\tilde{B}}$ is neglected in the following; i.e. only contributions from $\mathcal{O}_{\tilde{B}B}$ and $\mathcal{O}_{\tilde{W}W}$ are taken into account.

After electroweak symmetry breaking in the unitary gauge the effective Lagrangian in the mass basis of Higgs boson *H*, photon *A* and weak gauge bosons *Z* and $W^\pm$ can be written, e.g. as in Ref. [26]:

3 $$\begin{aligned} \mathcal{L}_{\mathrm {eff}}= & {} \mathcal{L}_{\mathrm {SM}} + \tilde{g}_{HAA} H \tilde{A}_{\mu \nu }{A}^{\mu \nu } + \tilde{g}_{HAZ} H \tilde{A}_{\mu \nu }{Z}^{\mu \nu }\nonumber \\&\,+ \tilde{g}_{HZZ} H \tilde{Z}_{\mu \nu }{Z}^{\mu \nu } + \tilde{g}_{HWW} H \tilde{W}^+_{\mu \nu }{W}^{-\mu \nu }. \end{aligned}$$

Only two of the four couplings $\tilde{g}_{HVV}$ ($V=W^\pm ,Z,\gamma$) are independent due to constraints imposed by U(1)$_Y$ and SU(2)$_{I_W,\mathrm{L}}$ invariance. They can be expressed in terms of two dimensionless couplings $\tilde{d}$ and $\tilde{d}_B$ as:

4 $$\begin{aligned} \tilde{g}_{HAA} = \frac{g}{2 m_W} (\tilde{d} \sin ^2 \theta _W + \tilde{d}_B \cos ^2 \theta _W)&\tilde{g}_{HAZ} = \frac{g}{2 m_W} \sin 2\theta _W (\tilde{d} -\tilde{d}_B)\end{aligned}$$

5 $$\begin{aligned} \tilde{g}_{HZZ} = \frac{g}{2 m_W} (\tilde{d} \cos ^2 \theta _W + \tilde{d}_B \sin ^2 \theta _W)&\tilde{g}_{HWW} = \frac{g}{m_W} \tilde{d}. \end{aligned}$$

Hence in general *WW*, *ZZ*, $Z\gamma$ and $\gamma \gamma$ fusion contribute to VBF production. The relations between $\tilde{d}$ and $f_{\tilde{W}W}$, and $\tilde{d}_B$ and $f_{\tilde{B}B}$ are given by:

6 $$\begin{aligned} \tilde{d} = - \frac{m_W^2}{\Lambda ^2} f_{\tilde{W}W}\quad \tilde{d}_B = - \frac{m_W^2}{\Lambda ^2} \tan ^2 \theta _W f_{\tilde{B}B}. \end{aligned}$$

As the different contributions from the various electroweak gauge-boson fusion processes cannot be distinguished experimentally with the current available dataset, the arbitrary choice $\tilde{d} = \tilde{d}_B$ is adopted. This yields the following relation for the $\tilde{g}_{HVV}$:

7 $$\begin{aligned} \tilde{g}_{HAA} = \tilde{g}_{HZZ} = \frac{1}{2} \tilde{g}_{HWW} = \frac{g}{2 m_W} \tilde{d}\quad {\mathrm {and}} \quad \tilde{g}_{HAZ} = 0 \, . \end{aligned}$$

The parameter $\tilde{d}$ is related to the parameter $\hat{\kappa }_W = \tilde{\kappa }_W/ \kappa _{\mathrm {SM}} \tan \alpha$ used in the investigation of CP properties in the decay $H\rightarrow WW$ [15] via $\tilde{d} = - \hat{\kappa }_W$. The choice $\tilde{d} = \tilde{d}_B$ yields $\hat{\kappa }_W = \hat{\kappa }_Z$ as assumed in the combination of the $H\rightarrow WW$ and $H\rightarrow ZZ$ decay analyses [15].

The effective Lagrangian yields the following Lorentz structure for each vertex in the Higgs bosons coupling to two identical or charge-conjugated electroweak gauge bosons $HV(p_1)V(p_2)$ ($V=W^{\pm},Z,\gamma$), with $p_{1,2}$ denoting the momenta of the gauge bosons:

8 $$\begin{aligned}&T^{\mu \nu } (p_1,p_2) = \sum _{V=W,Z} \frac{2m^{2}_{V}}{v} g^{\mu \nu } + \sum _{V=W,Z,\gamma }\frac{2g}{m_W} \tilde{d} ~ \varepsilon ^{\mu \nu \rho \sigma } p_{1\rho } p_{2\sigma }. \end{aligned}$$

The first terms ($\propto g^{\mu \nu }$) are CP-even and describe the SM coupling structure, while the second terms ($\propto \varepsilon ^{\mu \nu \rho \sigma } p_{1\rho } p_{2\sigma }$) are CP-odd and arise from the CP-odd dimension-six operators. The choice $\tilde{d} = \tilde{d}_B$ gives the same coefficients multiplying the CP-odd structure for $HW^+W^-$, *HZZ* and $H\gamma \gamma$ vertices and a vanishing coupling for the $HZ\gamma$ vertex.

The matrix element $\mathcal {M}$ for VBF production is the sum of a CP-even contribution $\mathcal {M}_{\text {SM}}$ from the SM and a CP-odd contribution $\mathcal {M}_{\text {CP-odd}}$ from the dimension-six operators considered:

9 $$\begin{aligned} \mathcal {M} =\mathcal {M}_{\text {SM}}+\tilde{d}\cdot \mathcal {M}_{\text {CP-odd}}. \end{aligned}$$

The differential cross section or squared matrix element has three contributions:

10 $$\begin{aligned} |\mathcal {M}|^{2}=|\mathcal {M}_{\text {SM}}|^{2}+ \tilde{d}\cdot 2 \mathrm{Re}(\mathcal {M}_{\text {SM}}^{*}\mathcal {M}_{\text {CP-odd}}) + \tilde{d}^{2}\cdot |\mathcal {M}_{\text {CP-odd}}|^{2} \, . \end{aligned}$$

The first term $|\mathcal {M}_{\text {SM}}|^{2}$ and third term $\tilde{d}^{2}\cdot |\mathcal {M}_{\text {CP-odd}}|^{2}$ are both CP-even and hence do not yield a source of CP violation. The second term $\tilde{d}\cdot 2 \mathrm{Re}(\mathcal {M}_{\text {SM}}^{*}\mathcal {M}_{\text {CP-odd}})$, stemming from the interference of the two contributions to the matrix element, is CP-odd and is a possible new source of CP violation in the Higgs sector. The interference term integrated over a CP-symmetric part of phase space vanishes and therefore does not contribute to the total cross section and observed event yield after applying CP-symmetric selection criteria. The third term increases the total cross section by an amount quadratic in $\tilde{d}$, but this is not exploited in the analysis presented here.

## Test of CP invariance and *Optimal Observable*

Tests of CP invariance can be performed in a completely model-independent way by measuring the mean value of a CP-odd observable $\langle \mathcal {O}_{\text {CP}} \rangle$. If CP invariance holds, the mean value has to vanish $\langle \mathcal {O}_{\text {CP}} \rangle = 0$. An observation of a non-vanishing mean value would be a clear sign of CP violation. A simple CP-odd observable for Higgs boson production in VBF, the “signed” difference in the azimuthal angle between the two tagging jets $\Delta \phi _{jj}$, was suggested in Ref. [22] and is formally defined as:

11 $$\begin{aligned} \epsilon _{\mu \nu \rho \sigma } b_+^\mu p_+^\nu b_-^\rho p_-^\sigma = 2 p_\mathrm{T+} p_\mathrm{T-} \sin (\phi _+ - \phi _-) = 2 p_\mathrm{T+} p_\mathrm{T-} \sin \Delta \phi _{jj} \, . \end{aligned}$$

Here $b_+^\mu$ and $b_-^\mu$ denote the normalised four-momenta of the two proton beams, circulating clockwise and anti-clockwise, and $p_+^\mu$ ($\phi _+$) and $p_-^\mu$ ($\phi _-$) denote the four-momenta (azimuthal angles) of the two tagging jets, where $p_+$ ($p_-$) points into the same detector hemisphere as $b_+^\mu$ ($b_-^\mu$). This ordering of the tagging jets by hemispheres removes the sign ambiguity in the standard definition of $\Delta \phi _{jj}$.

The final state consisting of the Higgs boson and the two tagging jets can be characterised by seven phase-space variables while assuming the mass of the Higgs boson, neglecting jet masses and exploiting momentum conservation in the plane transverse to the beam line. The concept of the *Optimal Observable* combines the information of the high-dimensional phase space in a single observable, which can be shown to have the highest sensitivity for small values of the parameter of interest and neglects contributions proportional to $\tilde{d}^2$ in the matrix element. The method was first suggested for the estimation of a single parameter using the mean value only [17] and via a maximum-likelihood fit to the full distribution [18] using the so-called *Optimal Observable* of first order. The extension to several parameters and also exploiting the matrix-element contributions quadratic in the parameters by adding an *Optimal Observable* of second order was introduced in Refs. [19, 27, 28]. The technique has been applied in various experimental analyses, e.g. Refs. [15, 29–39].

The analysis presented here uses only the first-order *Optimal Observable* $\mathcal {\,OO\,}$ (called *Optimal Observable* below) for the measurement of $\tilde{d}$ via a maximum-likelihood fit to the full distribution. It is defined as the ratio of the interference term in the matrix element to the SM contribution:

12 $$\begin{aligned} \mathcal {OO} =\frac{2 \mathrm{Re}(\mathcal {M}_{\text {SM}}^{*}\mathcal {M}_{\text {CP-odd}})}{|\mathcal {M}_{\text {SM}}|^{2}} \, . \end{aligned}$$

**Fig. 1:**
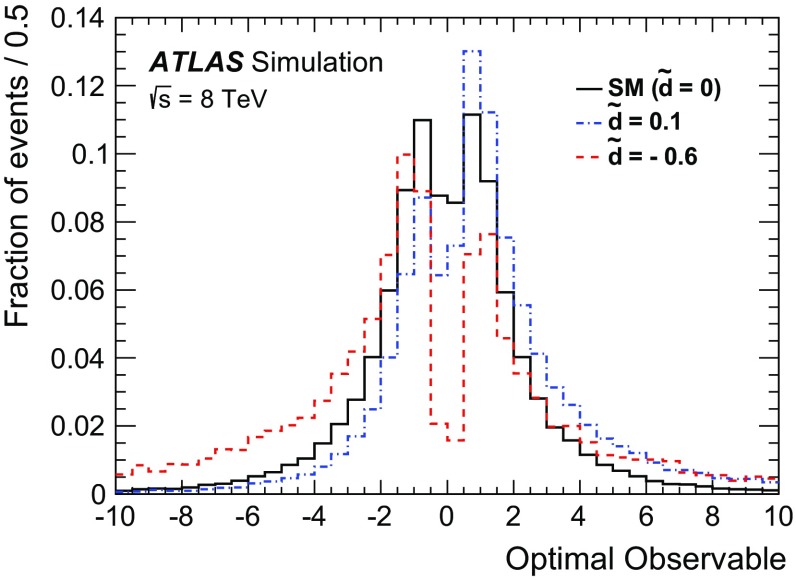
Distribution of the *Optimal Observable* at parton-level for two arbitrary $\tilde{d}$ values. The SM sample was generated using MadGraph5_aMC@NLO [40] (see Sect. 5) at leading order, and then reweighted to different $\tilde{d}$ values. Events are chosen such that there are at least two outgoing partons with $p_{\mathrm {T}}> 25 \,\mathrm{GeV}$, $|\eta | < 4.5$, large invariant mass ($m(p_1,p_2) > 500 \,\mathrm{GeV}$) and large pseudorapidity gap ($\Delta \eta (p_1,p_2) > 2.8$ )

Figure 1 shows the distribution of the *Optimal Observable*, at parton level both for the SM case and for two non-zero $\tilde{d}$ values, which introduce an asymmetry into the distribution and yield a non-vanishing mean value.

The values of the leading-order matrix elements needed for the calculation of the *Optimal Observable* are extracted from HAWK [41–43]. The evaluation requires the four-momenta of the Higgs boson and the two tagging jets. The momentum fraction $x_{1}$ ($x_{2}$) of the initial-state parton from the proton moving in the positive (negative) *z*-direction can be derived by exploiting energy–momentum conservation from the Higgs boson and tagging jet four-momenta as:

13 $$\begin{aligned} x_{1/2}^{\text {reco}} = \frac{m_{Hjj}}{\sqrt{s}}\mathrm {e}^{\pm y_{Hjj}} ~~~~~~ \end{aligned}$$

where $m_{\text {Hjj}}$ ($y_{\text {Hjj}}$) is the invariant mass (rapidity) obtained from the vectorially summed four-momenta of the tagging jets and the Higgs boson. Since the flavour of the initial- and final-state partons cannot be determined experimentally, the sum over all possible flavour configurations $ij \rightarrow kl H$ weighted by the CT10 leading-order parton distribution functions (PDFs) [44] is calculated separately for the matrix elements in the numerator and denominator:

14 $$\begin{aligned} 2 \mathrm{Re}(\mathcal {M}_{\text {SM}}^{*}\mathcal {M}_{\text {CP-odd}})= & {} \sum _{i,j,k,l} f_i(x_1) f_j(x_2) 2 \mathrm{Re}((\mathcal {M}_{\text {SM}}^{ij \rightarrow kl H})^*\mathcal {M}_{\text {CP-odd}}^{ij \rightarrow kl H})\end{aligned}$$

15 $$\begin{aligned} |\mathcal {M}_{\mathrm {SM}}|^2= & {} \sum _{i,j,k,l} f_i(x_1) f_j(x_2) |\mathcal {M}_{\mathrm {SM}}^{ij \rightarrow kl H}|^2\, . \end{aligned}$$

## The ATLAS detector

The ATLAS detector [45] is a multi-purpose detector with a cylindrical geometry.^1^ It comprises an inner detector (ID) surrounded by a thin superconducting solenoid, a calorimeter system and an extensive muon spectrometer in a toroidal magnetic field. The ID tracking system consists of a silicon pixel detector, a silicon microstrip detector, and a transition radiation tracker. It provides precise position and momentum measurements for charged particles and allows efficient identification of jets containing *b*-hadrons (*b*-jets) in the pseudorapidity range $|\eta |<2.5$. The ID is immersed in a 2 T axial magnetic field and is surrounded by high-granularity lead/liquid-argon sampling electromagnetic calorimeters which cover the pseudorapidity range $|\eta |< 3.2$. A steel/scintillator tile calorimeter provides hadronic energy measurements in the central pseudorapidity range ($|\eta |< 1.7$). In the forward regions ($1.5< |\eta | < 4.9$), the system is complemented by two end-cap calorimeters using liquid argon as active material and copper or tungsten as absorbers. The muon spectrometer surrounds the calorimeters and consists of three large superconducting eight-coil toroids, a system of tracking chambers, and detectors for triggering. The deflection of muons is measured in the region $|\eta |< 2.7$ by three layers of precision drift tubes, and cathode strip chambers in the innermost layer for $|\eta | > 2.0$. The trigger chambers consist of resistive plate chambers in the barrel ($|\eta | < 1.05$) and thin-gap chambers in the end-cap regions ($1.05<|\eta |<2.4$).

A three-level trigger system [46] is used to select events. A hardware-based Level-1 trigger uses a subset of detector information to reduce the event rate to 75 kHz or less. The rate of accepted events is then reduced to about 400 Hz by two software-based trigger levels, named Level-2 and the Event Filter.

## Simulated samples

Background and signal events are simulated using various Monte Carlo (MC) event generators, as summarised in Table 1. The generators used for the simulation of the hard-scattering process and the model used for the simulation of the parton shower, hadronisation and underlying-event activity are listed. In addition, the cross-section values to which the simulation is normalised and the perturbative order in QCD of the respective calculations are provided.

**Table 1:** MC event generators used to model the signal and the background processes at $\sqrt{s}=8\,\mathrm{TeV}$

Signal	MC generator	$\sigma \times \mathcal{B}$ [pb]		
		$$\sqrt{s}=8~\,\mathrm{TeV} $$		
VBF, $H\rightarrow \tau \tau$	Powheg-Box [47–50] Pythia8 [51]	0.100	(N)NLO	[41, 42, 52–54]
VBF, $H\rightarrow WW$	same as for $H\rightarrow \tau \tau$ signal	0.34	(N)NLO	[41, 42, 52–54]
Background	MC generator	$\sigma \times \mathcal{B}$ [pb]		
		$$\sqrt{s}=8~\,\mathrm{TeV} $$		
$W (\rightarrow \ell \nu$), ($\ell = e, \mu , \tau )$	Alpgen [55] + Pythia8	36,800	NNLO	[56, 57]
$Z/\gamma ^{*}(\rightarrow \ell \ell )$,	Alpgen + Pythia8	3910	NNLO	[56, 57]
60 $\,\mathrm{GeV}$ $<m_{\ell \ell }<2$ $\,\mathrm{TeV}$				
$Z/\gamma ^{*}(\rightarrow \ell \ell )$,	Alpgen + Herwig [58]	13,000	NNLO	[56, 57]
10 $\,\mathrm{GeV}$ $<m_{\ell \ell }<60$ $\,\mathrm{GeV}$				
VBF $Z/\gamma ^{*}$($\rightarrow \ell \ell$)	Sherpa [59]	1.1	LO	[59]
$t\bar{t}$	Powheg-Box + Pythia8	253$^{\dagger }$	NNLO + NNLL	[60–65]
Single top : *Wt*	Powheg-Box + Pythia8	22$^{\dagger }$	NNLO	[66]
Single top : *s*-channel	Powheg-Box + Pythia8	5.6$^{\dagger }$	NNLO	[67]
Single top : *t*-channel	AcerMC [68] + Pythia6 [69]	87.8$^{\dagger }$	NNLO	[70]
$q\bar{q} \rightarrow WW$	Alpgen+Herwig	54$^{\dagger }$	NLO	[71]
$gg \rightarrow WW$	gg2WW [72] + Herwig	1.4$^{\dagger }$	NLO	[72]
*WZ*, *ZZ*	Herwig	30$^{\dagger }$	NLO	[71]
ggF, $H\rightarrow \tau \tau$	HJ MINLO [73, 74] + Pythia8	1.22	NNLO + NNLL	[54, 75–80]
ggF, $H\rightarrow WW$	Powheg-Box [81] + Pythia8	4.16	NNLO + NNLL	[54, 75–80]

All Higgs boson events are generated assuming $m_{H} = 125 \,\mathrm{GeV}$. The cross sections times branching fractions ($\sigma \times \mathcal{B}$) used for the normalisation of some processes (many of these are subsequently normalised to data) are included in the last column together with the perturbative order of the QCD calculation. For the signal processes the $H\rightarrow \tau \tau$ and $H\rightarrow WW$ SM branching ratios are included, and for the *W* and $Z/\gamma ^{*}$ background processes the branching ratios for leptonic decays ($\ell = e, \mu ,\tau$) of the bosons are included. For all other background processes, inclusive cross sections are quoted (marked with a $\dagger$)

All the background samples used in this analysis are the same as those employed in Ref. [20], except the ones used to simulate events with the Higgs boson produced via gluon fusion and decaying into the $\tau \tau$ final state. The Higgs-plus-one-jet process is simulated at NLO accuracy in QCD with Powheg-Box [47–49, 73], with the MINLO feature  [74] applied to include Higgs-plus-zero-jet events at NLO accuracy. This sample is referred to as HJ MINLO. The Powheg-Box event generator is interfaced to Pythia8 [51], and the CT10 [44] parameterisation of the PDFs is used. Higgs boson events produced via gluon fusion and decaying into the $W^{+}W^{-}$ final state, which are a small component of the background, are simulated, as in Ref. [20], with Powheg [47–49, 81] interfaced to Pythia8 [51]. For these simulated events, the shape of the generated $p_{\mathrm {T}}$ distribution is matched to a NNLO + NNLL calculation HRes2.1 [82, 83] in the inclusive phase space. Simultaneously, for events with two or more jets, the Higgs boson $p_{\mathrm {T}}$ spectrum is reweighted to match the MINLO HJJ predictions [84]. The overall normalisation of the gluon fusion process (ggF) is taken from a calculation at next-to-next-to-leading order (NNLO) [75–80] in QCD, including soft-gluon resummation up to next-to-next-to-leading logarithm terms (NNLL) [85]. Next-to-leading-order (NLO) electroweak (EW) corrections are also included [86, 87]. Higgs boson events produced via VBF, with SM couplings, are also simulated with Powheg interfaced with Pythia8 (see Table 1 and Ref. [20]).

Production by VBF is normalised to a cross section calculated with full NLO QCD and EW corrections [41, 42, 52] with an approximate NNLO QCD correction applied [53]. The NLO EW corrections for VBF production depend on the $p_{\mathrm {T}}$ of the Higgs boson, and vary from a few percent at low $p_{\mathrm {T}}$ to $\sim 20\%$ at $p_{\mathrm {T}}$ = 300 $\,\mathrm{GeV}$  [88]. The $p_{\mathrm {T}}$ spectrum of the VBF-produced Higgs boson is therefore reweighted, based on the difference between the Powheg-Box+Pythia calculation and the Hawk [41–43] calculation which includes these corrections.

In the case of VBF-produced Higgs boson events in the presence of anomalous couplings in the *HVV* vertex, the simulated samples are obtained by applying a matrix element (ME) reweighting method to the VBF SM signal sample. The weight is defined as the ratio of the squared ME value for the VBF process associated with a specific amount of CP mixing (measured in terms of $\tilde{d}$) to the SM one. The inputs needed for the ME evaluation are the flavour of the incoming partons, the four-momenta and the flavour of the two or three final-state partons and the four-momentum of the Higgs boson. The Bjorken *x* values of the initial-state partons can be calculated from energy–momentum conservation. The leading-order ME from HAWK [41–43] is used for the $2\rightarrow 2+H$ or $2\rightarrow 3+H$ process separately. This reweighting procedure is validated against samples generated with MadGraph5_aMC@NLO [40]. As described in Ref. [89], MadGraph5_aMC@NLO can simulate VBF production with anomalous couplings at next-to-leading order. The reweighting procedure proves to be a good approximation to a full next-to-Leading description of the BSM process.

In the case of the $H\rightarrow WW$ sample, if CP violation exists in the *HVV* coupling, it would affect both the VBF production and the *HWW* decay vertex. It was verified that the shape of the *Optimal Observable* distribution is independent of any possible CP violation in the $H\rightarrow WW$ decay vertex and that it is identical for $H\rightarrow WW$ and $H\rightarrow \tau \tau$ decays. Hence the same reweighting is applied for VBF-produced events with $H\rightarrow WW$ and $H\rightarrow \tau \tau$ decays.

For all samples, a full simulation of the ATLAS detector response [90] using the Geant4 program [91] was performed. In addition, multiple simultaneous minimum-bias interactions are simulated using the AU2 [92] parameter tuning of Pythia8. They are overlaid on the simulated signal and background events according to the luminosity profile of the recorded data. The contributions from these pile-up interactions are simulated both within the same bunch crossing as the hard-scattering process and in neighbouring bunch crossings. Finally, the resulting simulated events are processed through the same reconstruction programs as the data.

## Analysis

After data quality requirements, the integrated luminosity of the $\sqrt{s}=8\,\mathrm{TeV}$ dataset used is 20.3 fb$^{-1}$. The triggers, event selection, estimation of background contributions and systematic uncertainties closely follow the analysis in Ref. [20]. In the following a short description of the analysis strategy is given; more details are given in that reference.

Depending on the reconstructed decay modes of the two $\tau$ leptons (leptonic or hadronic), events are separated into the dileptonic ($\tau _{\mathrm {lep}}\tau _{\mathrm {lep}}$) and semileptonic ($\tau _{\mathrm {lep}}\tau _{\mathrm {had}}$) channels. Following a channel-specific preselection, a VBF region is selected by requiring at least two jets with $p_{\mathrm {T}}^{j_1}>$ 40 $\,\mathrm{GeV}$ (50 $\,\mathrm{GeV}$) and $p_{\mathrm {T}}^{j_2}>30$ $\,\mathrm{GeV}$ and a pseudorapidity separation $\Delta \eta (j_1,j_2) > 2.2$ (3.0) in the $\tau _{\mathrm {lep}}\tau _{\mathrm {lep}}$ ($\tau _{\mathrm {lep}}\tau _{\mathrm {had}}$) channel. Events with *b*-tagged jets are removed to suppress top-quark backgrounds.

**Fig. 2:**
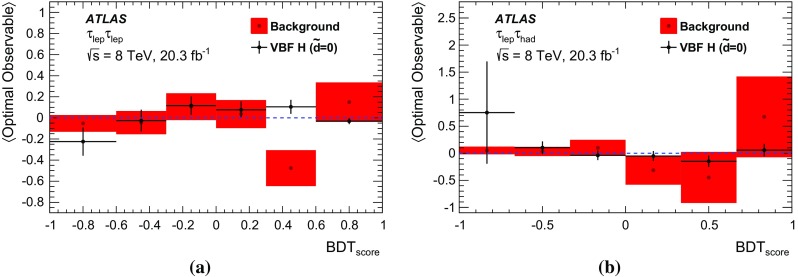
Mean of the *Optimal Observable* as a function of the BDT$_{\text {score}}$ for the SM signal (*black dots with error bars*) and for the sum of all background processes (*filled red area*), for the **a** $\tau _{\mathrm {lep}}\tau _{\mathrm {lep}}$ and **b** $\tau _{\mathrm {lep}}\tau _{\mathrm {had}}$ channel. The signal and background model is in agreement with the hypothesis of no bias from the BDT score

Inside the VBF region, boosted decision trees (BDT)^2^ are utilised for separating Higgs boson events produced via VBF from the background (including other Higgs boson production modes). The final signal region in each channel is defined by the events with a BDT$_{\text {score}}$ value above a threshold of 0.68 for $\tau _{\mathrm {lep}}\tau _{\mathrm {lep}}$ and 0.3 for $\tau _{\mathrm {lep}}\tau _{\mathrm {had}}$. The efficiency of this selection, with respect to the full VBF region, is 49% (51%) for the signal and 3.6% (2.1%) for the sum of background processes for the $\tau _{\mathrm {lep}}\tau _{\mathrm {lep}}$ ($\tau _{\mathrm {lep}}\tau _{\mathrm {had}}$) channel. A non-negligible number of events from VBF-produced $H\rightarrow WW$ events survive the $\tau _{\mathrm {lep}}\tau _{\mathrm {lep}}$ selection: they amount to 17% of the overall VBF signal in the signal region. Their contribution is entirely negligible in the $\tau _{\mathrm {lep}}\tau _{\mathrm {had}}$ selection. Inside each signal region, the *Optimal Observable* is then used as the variable with which to probe for CP violation. The BDT$_{\text {score}}$ does not affect the mean of the *Optimal Observable*, as can be seen in Fig. 2.

**Fig. 3:**
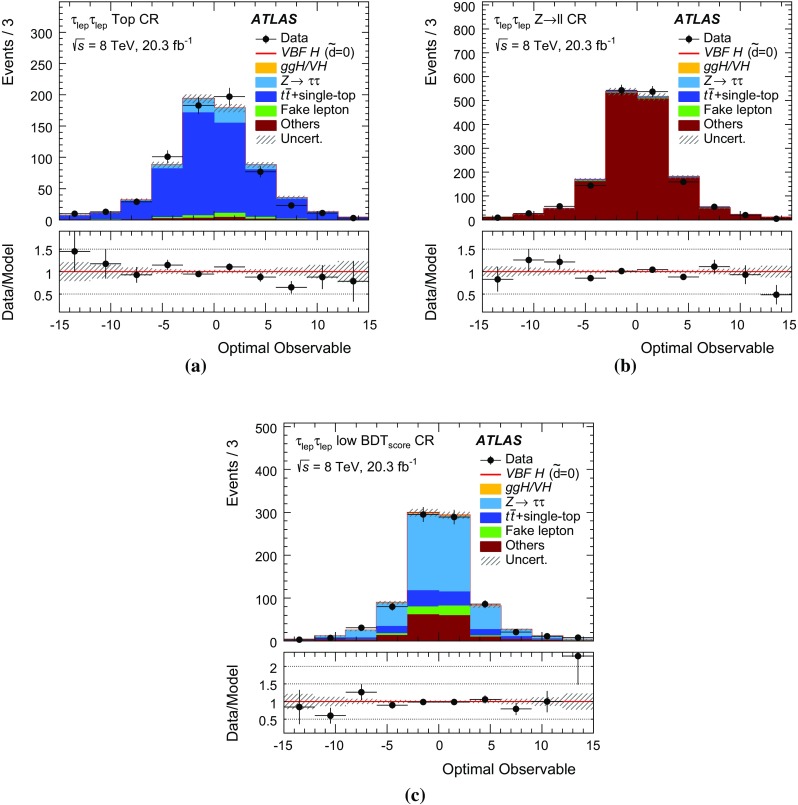
Distributions of the *Optimal Observable* for the $\tau _{\mathrm {lep}}\tau _{\mathrm {lep}}$ channel in the **a** top-quark control region (CR), **b** $Z\rightarrow \ell \ell$ CR, and **c** low-BDT$_{\text {score}}$ CR. The CR definitions are given in the text. These figures use background predictions before the global fit defined in Sect. 7. The “Other” backgrounds include diboson and $Z\rightarrow \ell \ell$. Only statistical uncertainties are shown

**Fig. 4:**
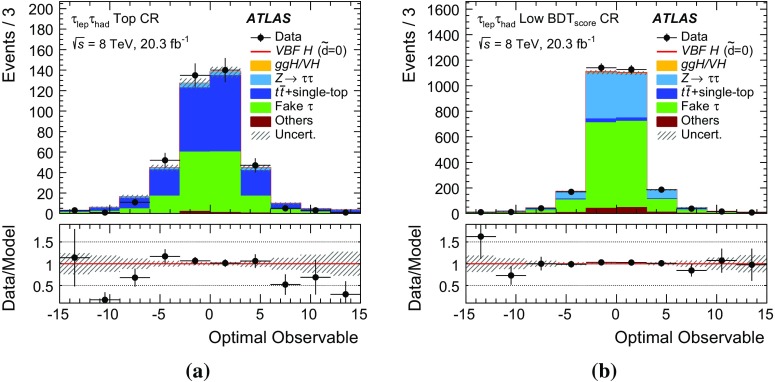
Distributions of the *Optimal Observable* for the $\tau _{\mathrm {lep}}\tau _{\mathrm {had}}$ channel in the **a** top-quark control region (CR) and **b** low-BDT$_{\text {score}}$ CR. The CR definitions are given in the text. These figures use background predictions before the global fit defined in Sect. 7. The “Other” backgrounds include diboson and $Z\rightarrow \ell \ell$. Only statistical uncertainties are shown

The modelling of the *Optimal Observable* distribution for various background processes is validated in dedicated control regions. The top-quark control regions are defined by the same cuts as the corresponding signal region, but inverting the veto on *b*-tagged jets and not applying the selection on the BDT$_{\text {score}}$ (in the $\tau _{\mathrm {lep}}\tau _{\mathrm {had}}$ channel a requirement of the transverse mass^3^ $m_\mathrm {T}>40$ GeV is also applied). In the $\tau _{\mathrm {lep}}\tau _{\mathrm {lep}}$ channel a $Z\rightarrow \ell \ell$ control region is obtained by requiring two same-flavour opposite-charge leptons, the invariant mass of the two leptons to be $80< m_{\ell \ell } < 100~\,\mathrm{GeV}$, and no BDT$_{\text {score}}$ requirement, but otherwise applying the same requirements as for the signal region. These regions are also used to normalise the respective background estimates using a global fit described in the next section. Finally, an additional region is defined for each channel, called the low-BDT$_{\text {score}}$ control region, where a background-dominated region orthogonal to the signal region is selected by requiring the BDT$_{\text {score}}$ to be less than 0.05 for $\tau _{\mathrm {lep}}\tau _{\mathrm {lep}}$ and less than 0.3 for $\tau _{\mathrm {lep}}\tau _{\mathrm {had}}$. The distribution of the *Optimal Observable* in these regions is shown in Figs. 3 and  4, demonstrating the good description of the data by the background estimates.

The effect of systematic uncertainties on the yields in signal region and on the shape of the *Optimal Observable* is evaluated following the procedures and prescriptions described in Ref. [20]. An additional theoretical uncertainty in the shape of the *Optimal Observable* is included to account for the signal reweighting procedure described in Sect. 5. This is obtained from the small difference between the *Optimal Observable* distribution in reweighted samples, compared to samples with anomalous couplings directly generated with MadGraph5_aMC@NLO. While the analysis is statistically limited, the most important systematic uncertainties are found to arise from effects on the jet, hadronically decaying $\tau$ and electron energy scales; the most important theoretical uncertainty is due to the description of the underlying event and parton shower in the VBF signal sample.

## Fitting procedure

The best estimate of $\tilde{d}$ is obtained using a maximum-likelihood fit performed on the *Optimal Observable* distribution in the signal region for each decay channel simultaneously, with information from different control regions included to constrain background normalisations and nuisance parameters. The normalisation of the VBF $H\rightarrow \tau \tau$ and $H\rightarrow WW$ signal sample is left free in the fit, i.e. this analysis only exploits the shape of the *Optimal Observable* and does not depend on any possibly model-dependent information about the cross section of CP-mixing scenarios. The relative proportion of the two Higgs boson decay modes is assumed to be as in the SM. All other Higgs boson production modes are treated as background in this study and normalised to their SM expectation, accounting for the corresponding theoretical uncertainties.

A binned likelihood function $\mathcal {L}(\mathbf {x}; \mu , \varvec{\theta })$ is employed, which is a function of the data $\mathbf {x}$, the free-floating signal strength $\mu$, defined as the ratio of the measured cross section times branching ratio to the Standard Model prediction, and further nuisance parameters $\varvec{\theta }$. It relies on an underlying model of signal plus background, and it is defined as the product of Poisson probability terms for each bin in the distribution of the *Optimal Observable*. A set of signal templates corresponding to different values of the CP-mixing parameter $\tilde{d}$ is created by reweighting the SM VBF $H\rightarrow \tau \tau$ and $H\rightarrow WW$ signal samples, as described in Sect. 5. The likelihood function is then evaluated for each $\tilde{d}$ hypothesis using the corresponding signal template, while keeping the same background model. The calculation profiles the nuisance parameters to the best-fit values $\hat{\varvec{\theta }}$, including information about systematic uncertainties and normalisation factors, both of which affect the expected numbers of signal and background events.

After constructing the negative log-likelihood (NLL) curve by calculating the NLL value for each $\tilde{d}$ hypothesis, the approximate central confidence interval at 68% confidence level (CL) is determined from the best estimator $\hat{\tilde{d}}$, at which the NLL curve has its minimum value, by reading off the points at which $\Delta$NLL=NLL−NLL$_{\text {min}} = 0.5$. The expected sensitivity is determined using an Asimov dataset, i.e. a pseudo-data distribution equal to the signal-plus-background expectation for given values of $\tilde{d}$ and the parameters of the fit, in particular the signal strength $\mu$, and not including statistical fluctuations [93].

In both channels, a region of low BDT$_{\text {score}}$ is obtained as described in the preceding section. The distribution of the BDT$_{\text {score}}$ itself is fitted in this region, which has a much larger number of background events than the signal region, allowing the nuisance parameters to be constrained by the data. This region provides the main constraint on the $Z\rightarrow \tau \tau$ normalisation, which is free to float in the fit. The event yields from the top-quark (in $\tau _{\mathrm {lep}}\tau _{\mathrm {lep}}$ and $\tau _{\mathrm {lep}}\tau _{\mathrm {had}}$) and $Z\rightarrow \ell \ell$ (in $\tau _{\mathrm {lep}}\tau _{\mathrm {lep}}$ only) control regions defined in the previous section are also included in the fit, to constrain the respective background normalisations, which are also left free in the fit.

**Fig. 5:**
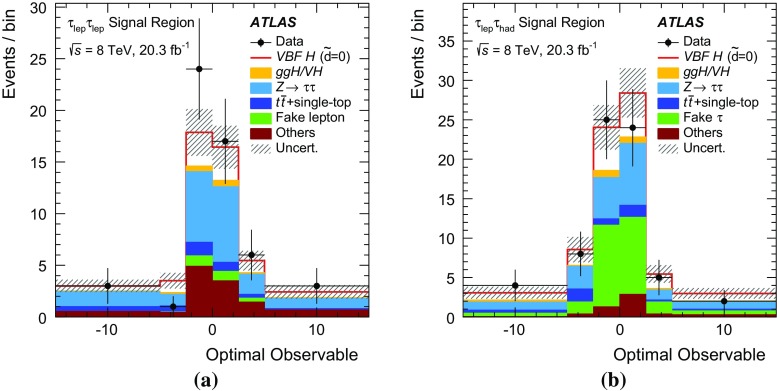
Distributions of the *Optimal Observable* in the signal region for the **a** $\tau _{\mathrm {lep}}\tau _{\mathrm {lep}}$ and **b** $\tau _{\mathrm {lep}}\tau _{\mathrm {had}}$ channel, after the global fit performed for the $\tilde{d}=0$ hypothesis. The best-fit signal strength is $\mu =1.55^{+0.87}_{-0.76}$. The “Other” backgrounds include diboson and $Z\rightarrow \ell \ell$. The error bands include all uncertainties

The distributions of the *Optimal Observable* in each channel are shown in Fig. 5, with the nuisance parameters, background and signal normalisation adjusted by the global fit performed for the $\tilde{d}=0$ hypothesis. Table 2 provides the fitted yields of signal and background events, split into the various contributions, in each channel. The number of events observed in data is also provided.

**Table 2:** Event yields in the signal region, after the global fit performed for the $\tilde{d}=0$ hypothesis. The errors include systematic uncertainties

Process	$\tau _{\mathrm {lep}}\tau _{\mathrm {lep}}$	$\tau _{\mathrm {lep}}\tau _{\mathrm {had}}$
Data	54	68
VBF $H\rightarrow \tau \tau$/*WW*	$9.8 \pm 2.1$	$16.7 \pm 4.1$
$Z\rightarrow \tau \tau$	$19.6 \pm 1.0$	$19.1 \pm 2.2$
Fake lepton/$\tau$	$2.3 \pm 0.3$	$24.1 \pm 1.5$
$t\bar{t}$ +single-top	$3.8 \pm 1.0$	$4.8 \pm 0.7$
Others	$11.5 \pm 1.7$	$5.3 \pm 1.6$
*ggH* / *VH*, $H\rightarrow \tau \tau /WW$	$1.6 \pm 0.2$	$2.5 \pm 0.7$
Sum of backgrounds	$38.9 \pm 2.3$	$55.8 \pm 3.3$

## Results

The mean value of the *Optimal Observable* for the signal is expected to be zero for a CP-even case, while there may be deviations in case of CP-violating effects. A mean value of zero is also expected for the background, as has been demonstrated. Hence, the mean value in data should also be consistent with zero if there are no CP-violating effects within the precision of this measurement. The observed values for the mean value in data inside the signal regions are $0.3\pm 0.5$ for $\tau _{\mathrm {lep}}\tau _{\mathrm {lep}}$ and $-0.3\pm 0.4$ for $\tau _{\mathrm {lep}}\tau _{\mathrm {had}}$, fully consistent with zero within statistical uncertainties and thus showing no hint of CP violation.

**Fig. 6:**
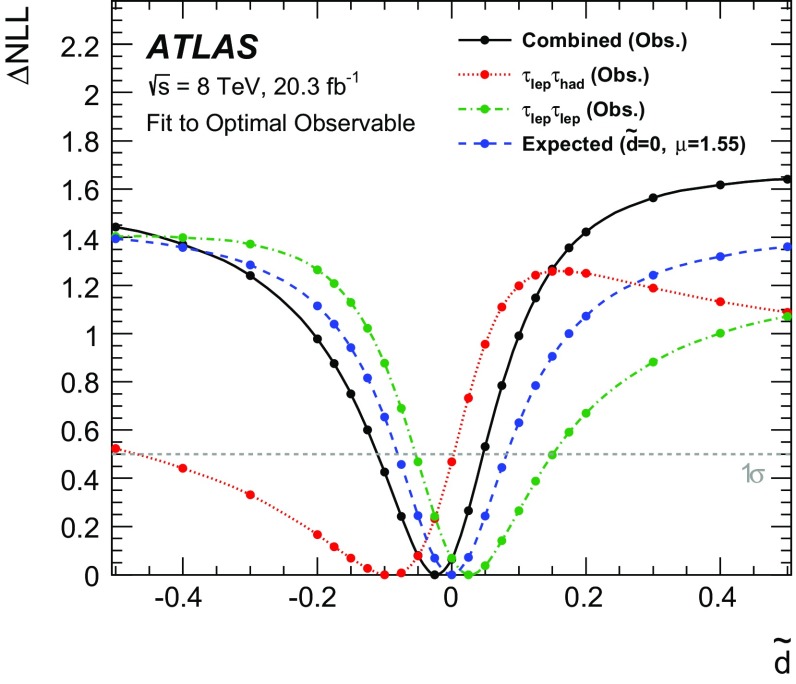
Observed and expected $\Delta$NLL as a function of the $\tilde{d}$ values defining the underlying signal hypothesis, for $\tau _{\mathrm {lep}}\tau _{\mathrm {lep}}$ (*green*), $\tau _{\mathrm {lep}}\tau _{\mathrm {had}}$ (*red*) and their combination (*black*). The best-fit values of all nuisance parameters from the combined fit at each $\tilde{d}$ point were used in all cases. An Asimov dataset with SM backgrounds plus pure CP-even VBF signal ($\tilde{d}=0$), scaled to the best-fit signal-strength value, was used to calculate the expected values, shown in *blue*. The *markers* indicate the points where an evaluation was made – the *lines* are only meant to guide the eye

**Fig. 7:**
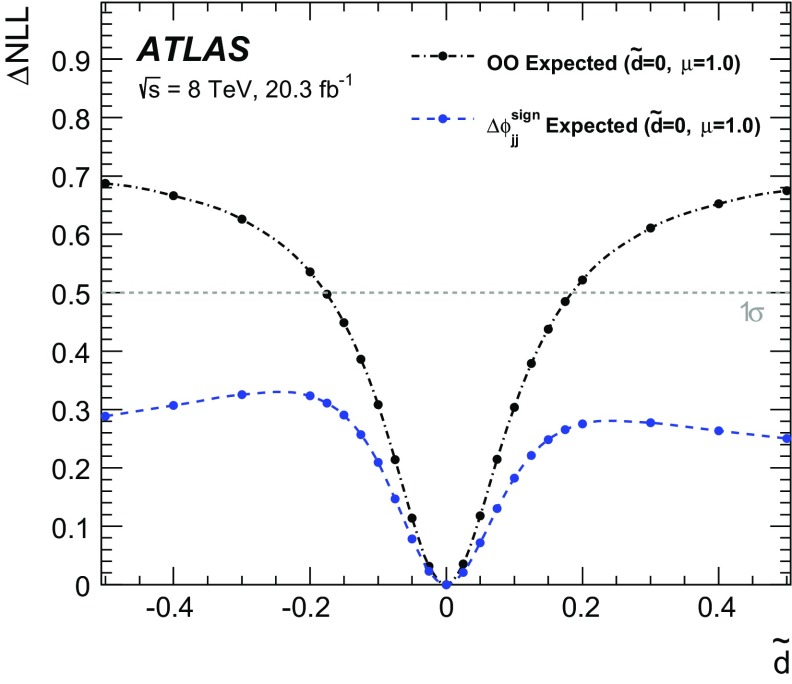
Expected $\Delta$NLL for the combination of both channels as a function of the $\tilde{d}$ values defining the underlying signal hypothesis when using the *Optimal Observable* (*black*) or the $\Delta \phi ^{\mathrm {sign}}_{jj}$ parameter (*blue*) as the final discriminating variable. An Asimov dataset with SM backgrounds plus pure CP-even VBF signal ($\tilde{d}=0$) scaled to the SM expectation was used to calculate the expected values in both cases. The markers indicate the points where an evaluation was made – the lines are only meant to guide the eye

**Fig. 8:**
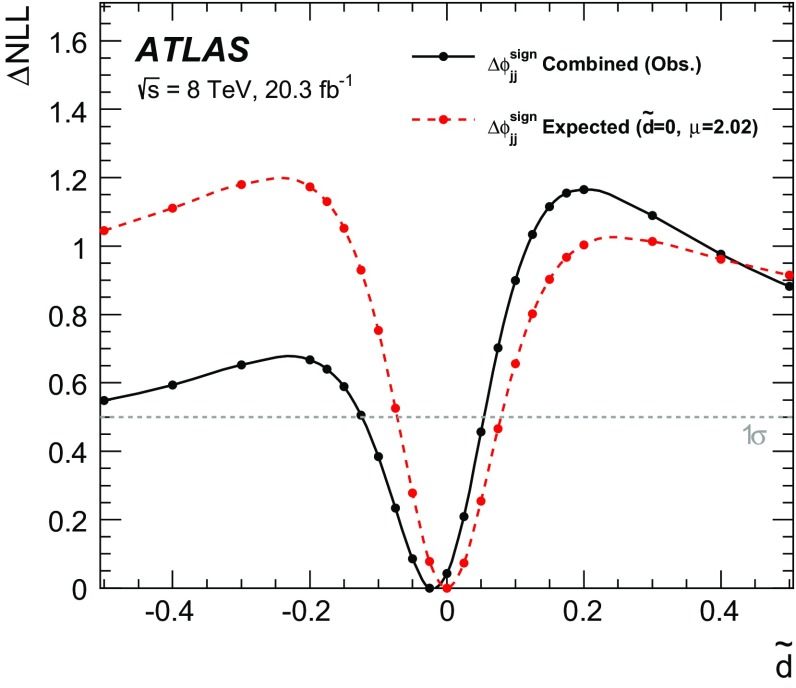
Observed (*black*) and expected (*red*) $\Delta$NLL for the combination of both channels as a function of the $\tilde{d}$ values defining the underlying signal hypothesis when using the $\Delta \phi ^{\mathrm {sign}}_{jj}$ parameter as the final discriminating variable. An Asimov dataset with SM backgrounds plus pure CP-even VBF signal ($\tilde{d}=0$), scaled to the best-fit value of the signal strength in the combined fit when using the $\Delta \phi ^{\mathrm {sign}}_{jj}$ parameter ($\mu =2.02^{+0.87}_{-0.77}$) was used to calculate the expected values. The markers indicate the points where an evaluation was made – the lines are only meant to guide the eye

As described in the previous section, the observed limit on CP-odd couplings is estimated using a global maximum-likelihood fit to the *Optimal Observable* distributions in data. The observed distribution of $\Delta$NLL as a function of the CP-mixing parameter $\tilde{d}$ for the individual channels separately, and for their combination, is shown in Fig. 6. The $\tau _{\mathrm {lep}}\tau _{\mathrm {lep}}$ and $\tau _{\mathrm {lep}}\tau _{\mathrm {had}}$ curves use the best-fit values of all nuisance parameters from the combined fit at each $\tilde{d}$ point. The expected curve is calculated assuming no CP-odd coupling, with the $H\rightarrow \tau \tau$ signal scaled to the signal-strength value ($\mu = 1.55^{+0.87}_{-0.76}$) determined from the fit for $\tilde{d}=0$. In the absence of CP violation the curve is expected to have a minimum at $\tilde{d}=0$. Since the first-order *Optimal Observable* used in the present analysis is only sensitive to small variations in the considered variable, for large $\tilde{d}$ values there is no further discrimination power and thus the $\Delta$NLL curve is expected to flatten out. The observed curve follows this behaviour and is consistent with no CP violation. The regions $\tilde{d}<-0.11$ and $\tilde{d}>0.05$ are excluded at 68% CL. The expected confidence intervals are $[-0.08 , 0.08]~([-0.18 , 0.18])$ for an assumed signal strength of $\mu =$ 1.55 (1.0). The constraints on the CP-mixing parameter $\tilde{d}$ based on VBF production can be directly compared to those obtained by studying the Higgs boson decays into vector bosons, as the same relation between the *HWW* and *HZZ* couplings as in Ref. [14, 15] is assumed. The 68% CL interval presented in this work is a factor 10 better than the one obtained in Ref. [15].

As a comparison, the same procedure for extracting the CP-mixing parameter $\tilde{d}$ was applied using the $\Delta \phi ^{\mathrm {sign}}_{jj}$ observable, previously proposed for this measurement and defined in Eq. 11, rather than the *Optimal Observable*. The expected $\Delta$NLL curves for a SM Higgs boson signal from the combination of both channels for the two CP-odd observables are shown in Fig. 7, allowing a direct comparison, and clearly indicate the better sensitivity of the *Optimal Observable*. The observed $\Delta$NLL curve derived from the $\Delta \phi ^{\mathrm {sign}}_{jj}$ distribution is also consistent with $\tilde{d}=0$, as shown in Fig. 8, along with the expectation for a signal with $\tilde{d}=0$ scaled to the best-fit signal-strength value ($\mu =2.02^{+0.87}_{-0.77}$).

## Conclusions

A test of CP invariance in the Higgs boson coupling to vector bosons has been performed using the vector-boson fusion production mode and the $H\rightarrow \tau \tau$ decay. The dataset corresponds to 20.3 $\mathrm{fb}^{-1}$of $\sqrt{s}$ = 8 $\,\mathrm{TeV}$ proton–proton collisions recorded by the ATLAS detector at the LHC. Event selection, background estimation and evaluation of systematic uncertainties are all very similar to the ATLAS analysis that provided evidence of the $H\rightarrow \tau \tau$ decay. An *Optimal Observable* is constructed and utilised, and is shown to provide a substantially better sensitivity than the variable traditionally proposed for this kind of study, $\Delta \phi ^{\mathrm {sign}}_{jj}$. No sign of CP violation is observed. Using only the dileptonic and semileptonic $H\rightarrow \tau \tau$ channels, and under the assumption $\tilde{d} = \tilde{d}_B$, values of $\tilde{d}$ less than $-0.11$ and greater than 0.05 are excluded at 68% CL.

This 68% CL interval is a factor of 10 better than the one previously obtained by the ATLAS experiment from Higgs boson decays into vector bosons. In contrast, the present analysis has no sensitivity to constrain a 95% CL interval with the dataset currently available – however larger data samples in the future and consideration of additional Higgs boson decay channels should make this approach highly competitive.

## Footnotes

1 ATLAS uses a right-handed coordinate system with its origin at the nominal interaction point (IP) in the centre of the detector and the *z*-axis along the beam pipe. The *x*-axis points from the IP to the centre of the LHC ring, and the *y*-axis points upward. Cylindrical coordinates $(r,\phi )$ are used in the transverse plane, $\phi$ being the azimuthal angle around the *z*-axis. The pseudorapidity is defined in terms of the polar angle $\theta$ as $\eta =-\ln \tan (\theta /2)$.

2 The same BDTs trained in the context of the analysis in Ref. [20] are used here, unchanged.

3 The transverse mass is defined as $m_{\mathrm {T}}=\sqrt{2p_{\mathrm {T}}^{\ell } \ E_{\mathrm {T}}^{\mathrm {miss}} \cdot (1-\cos {\Delta {\phi }})}$, where $\Delta {\phi }$ is the azimuthal separation between the directions of the lepton and the missing transverse momentum.
